# Association of diabetes mellitus and breast cancer in adult men and women: a cross-sectional survey

**DOI:** 10.1186/s12885-025-14689-6

**Published:** 2025-08-07

**Authors:** Rabiya Saroosh, Nazir Ahmad, Beenish Israr, Anum Nazir, Nizwa Itrat, Abdul Momin Rizwan Ahmad

**Affiliations:** 1 Department of Nutrition and Dietetics, The University of Faisalabad, Faisalabad, Pakistan; 2 Department of Nutritional Sciences, Government College University, Faisalabad, Faisalabad, Pakistan; 3 Faculty of Food, Nutrition and Home Sciences, Institute of Home Sciences, University of Agriculture, Faisalabad, Pakistan; 4 Department of Health Sciences, University of York, York, UK; 5 Department of Human Nutrition and Dietetics, NUST School of Health Sciences, National University of Sciences & Technology (NUST), Sector H-12, Islamabad, Pakistan

**Keywords:** Cross-sectional study, Body mass index, Postmenopausal status, Contraceptives, Diabetes mellitus, Associate risk factors

## Abstract

**Background:**

The co-occurrence of diabetes mellitus and breast cancer poses a significant global health challenge. Most research has focused on Western populations, with genetics, lifestyle, and environmental differences limiting generalizability to Pakistanis. This study aimed to fill this gap by investigating the diabetes-breast cancer association among Pakistani adults.

**Methods:**

A cross-sectional study was conducted from October 2023 to January 2024 at Pinum Cancer Hospital, Faisalabad. 400 participants were categorized as normal, diabetic, cancerous, and diabetic cancerous. Data were analyzed using SPSS with descriptive statistics and inferential tests at *p* < 0.05 significance.

**Results:**

Among 9,725 patients, 1.03% had concurrent diabetes and breast cancer. Females showed higher risk with a strong gender association (*P* < 0.0001) across groups. Significant associations were found for body weight (*P* < 0.009), BMI (*P* < 0.014), and fat distribution (*P* < 0.0001). Dietary factors such as fried fish (*P* < 0.001), red meat (*P* < 0.0001), processed food (*P* < 0.0001), and kitchen practices like overcooked oil (*P* < 0.003) were significant. Lifestyle factors including physical activity (*P* < 0.0001) and stress (*P* < 0.0001), environmental factors such as x-rays, and chemical exposure (*P* < 0.013) and female-specific factors like postmenopausal status (*P* < 0.0001) and contraceptive use (*p* < 0.032), and makeup (*P* < 0.0001) also showed significant association.

**Conclusion:**

Diabetes and breast cancer are strongly associated in Pakistani adults, influenced by clinical, dietary, lifestyle, and environmental factors. Personalized prevention and management strategies are crucial to improve treatment outcomes.

**Supplementary Information:**

The online version contains supplementary material available at 10.1186/s12885-025-14689-6.

## Background

Diabetes mellitus (DM) is a multifactorial disorder characterized by insulin resistance and insufficient insulin production, resulting in serious health complications [1]. Clinically diagnosed by fasting blood glucose > 126 mg/dL or an oral glucose tolerance test reading > 200 mg/dL, DM is usually associated with symptoms such as frequent urination, excessive thirst, and fatigue [2]. The global prevalence of DM among adults aged 20–79 is 10.5% (537 million people), rising sharply with age, from 4 to 6% in those aged 20–39 to 15–20% in the 40–59 age group, and exceeding 25% among adults 60–79 years [3]. According to the 10th edition of the IDF, 1 in 10 worldwide and over 3 in 4 adults with diabetes mellitus worldwide live in low- and middle-income countries. This number is expected to increase to 643 million by 2030 and 783 million by 2045, with the highest increase in low- and middle-income countries. In Pakistan, 30.8% of adults are affected, the third highest global prevalence, accounting for 17.5% of national deaths, indicating its significant public health burden [4].

This trend parallels rising cancer rates, leading to speculation about a possible direct link between diabetes and cancer, making it a major global health concern. The connection between the two conditions was first proposed in 1934 and has been studied extensively since then. Diabetes is now established as a recognized risk factor for many types of cancer, particularly breast cancer [5].

Breast cancer, a heterogeneous disease characterized by the uncontrolled proliferation of cells in breast tissue, is the most commonly diagnosed cancer in women worldwide [6]. It usually originates from the milk-producing glands (lobules) or the ducts connecting to the nipple. Early-stage breast cancer is often asymptomatic, but regular screening can significantly improve treatment outcomes. The most common clinical sign is a painless lump, which may spread to the underarm lymph nodes. Less common symptoms include breast pain, skin changes, and nipple discharge [7]. Globally, breast cancer is the fifth leading cause of cancer-related mortality, with more than 2 million new cases reported in 2018 and 1 in 8 women and ~ 1 in 1000 in males at lifetime risk of breast cancer [8, 9]. In South Asia, particularly Pakistan, it ranks first in Asia and second globally, with 90,000 new cases and 40,000 deaths annually, emphasizing the need for improved detection and treatment strategies [10].

Studies have shown that people with diabetes have an increased risk of developing various cancers, including breast cancer, with recent meta-analyses showing a 23% higher risk in women with diabetes. Preexisting diabetes is also associated with a 37% higher all-cause mortality risk and a 17% higher risk of breast cancer-specific mortality in female patients [11]. Insulin signaling regulates glucose uptake, glycogen synthesis, and lipid metabolism to maintain metabolic balance. Insulin binding activates IRS and Shc proteins, triggering PI3K-AKT and MAPK pathways for metabolism and cell growth. Insulin resistance disrupts these pathways, causing metabolic dysfunction. Additionally, insulin and IGFs activate HIF1 signaling, aiding cancer cell survival and progression under low oxygen. Combined risk factors like obesity, physical inactivity, and hyperinsulinemia contribute to this increased risk [12]. Furthermore, diabetes can exacerbate cancer progression, impair immune function, and reduce treatment efficacy, resulting in poorer outcomes [13].

Despite extensive research in Western populations, there is a notable gap in studies on the relationship between diabetes and breast cancer in Pakistan, a country with unique genetic, environmental, and lifestyle factors. Given the increasing prevalence of both conditions in Pakistan and the socio-cultural and healthcare challenges, the study aimed to investigate the concurrent prevalence of diabetes and breast cancer in Pakistani adults, identify diabetes-related risk factors for breast cancer development and progression and provide evidence-based recommendations to improve healthcare policies and clinical outcomes in the region.

## Methods

This cross-sectional descriptive study was conducted over four months (October 2023 to January 2024) at Pinum Cancer Hospital in Faisalabad, Pakistan.

**Participants: **Participants were selected using a simple random sampling technique to ensure diverse representation, reduce selection bias, and enhance the generalizability of the results. The sample size was calculated using the proportion formula.

$${\text{Sample size}}\,=\,{{\text{Z}}_{{1 - {\text{a/2}}}^{2}}}{\text{p}}\left( {{\text{1}} - {\text{p}}} \right)/{{\text{d}}^{\text{2}}}$$

Z1-a/2 = Is standard normal variate (at 5% type 1 error (*p* < 0.05) it is 1.96 and at 1% type 1 error (*p* < 0.01) it is 2.58). As in the majority of studies, p-values are considered significant below 0.05. Hence 1.96 was used in the formula.

p = Is the expected proportion of breast cancer patients who also have diabetes, based on a previous case-control study conducted in Pakistan (17.69%).

d = Is the desired margin of error or precision, set at 5% (0.05) [14].

This yielded a minimum sample size of approximately 255. To improve accuracy, facilitate subgroup analyses, and account for potential non-response or data loss, a larger sample of 400 individuals was recruited and divided equally into four groups of 100 each:

- **Normal group**: Individuals without diabetes or breast cancer.
- **Diabetic group**: Individuals diagnosed with diabetes mellitus (Table 1).
- **Cancerous group**: Individuals diagnosed with breast cancer (Table 2).
- **Diabetic Cancerous group**: Individuals with concurrent diabetes mellitus and breast cancer.

**Table 1:** Criteria for the diagnosis of diabetes mellitus (15)

• A1C ≥ 6.5% (48 m mol/mol)
OR
• 2-h plasma glucose ≥ 200 mg/dL (11.1 mmol/L)
OR
• Fasting plasma glucose ≥126 mg/dL (7.0 mmol/L)
OR
• Random plasma glucose ≥ 200 mg/dL (11.1 mmol/L)

**Table 2:** Criteria for the diagnosis of breast cancer (16)

• Diagnosis confirmed through mammography, ultrasound, or biopsy
OR
• Clinical staging based on the TNM (Tumor, Node, Metastasis) classification system
OR
• Histologically confirmed diagnosis of invasive breast carcinoma, including ductal carcinoma in situ (DCIS) and invasive ductal carcinoma (IDC)

Equal group sizes ensure sufficient statistical power for between-group comparisons, minimize bias from unequal sampling, and facilitate analysis of interactions between diabetes and breast cancer.

**Inclusion criteria**: This study included participants aged 20 to 79 years, which is in line with the International Diabetes Federation’s estimate that the prevalence of diabetes in this age group in Pakistan is significantly higher (30.8%) [15]. Both male and female individuals were enrolled in four categories: healthy, diabetes mellitus, breast cancer, and those with diabetes mellitus and breast cancer. Only participants who were diagnosed by a physician with complete medical reports and were able to complete the interview were eligible. The normal control group consisted of healthy males and females from the same geographic area, recruited from outpatient clinics, and the patient’s attendants.

**Exclusion criteria**: Participants younger than 20 years or older than 79 years, pre-diabetic, type 1 diabetes patients, current gestational diabetes patients, newly diagnosed type 2 diabetics, and patients with cancers other than breast cancer were excluded. Additionally, pregnant or lactating women, prior history of breast cancer or a diagnosis of breast cancer before diabetes mellitus were also excluded to reduce confounding factors. Individuals with incomplete medical records, or those unable to complete the interview, were also excluded to maintain data quality and reliability.

**Data collection: **Data were collected through structured face-to-face interviews conducted by trained interviewers in local languages (Urdu and Punjabi) to ensure clear communication and accurate information. Each interview lasted 20–25 min and used a pre-tested structured questionnaire that included demographics, medical history, dietary habits, kitchen practices, behavior, environmental factors, and female-specific characteristics. Additionally, qualitative methods such as observations and discussions complemented the data for a more comprehensive understanding.

**Statistical analysis**: Data were coded and entered into SPSS version 20 for efficient management and analysis. Rigorous data cleaning was performed to address missing data, outliers, and inconsistencies, ensuring accuracy and minimizing bias. Descriptive statistics were used to summarize demographic characteristics across study groups. Inferential analysis (chi-square for categorical and ANOVA for continuous variables) was used, with significance determined by p-values (< 0.05). This approach particularly assessed the prevalence of diabetes-related risk factors among adults with and without breast cancer, effectively addressing the study’s research objectives.

## Results

### Concurrent prevalence of diabetes mellitus and breast cancer

**Fig. 1:**
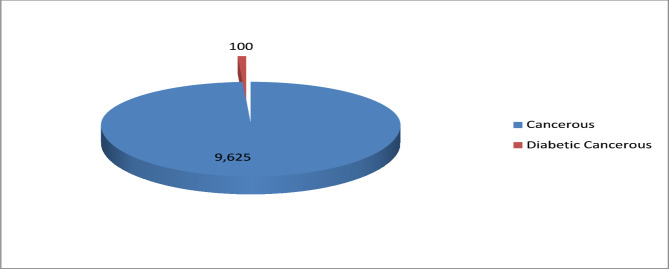
Concurrent prevalence of diabetes mellitus and breast cancer

During the four-month study period, the Oncology Department of Pinum Cancer Hospital, Faisalabad treated 9,725 patients, of whom 100 were diagnosed with both diabetes mellitus and breast cancer (Fig. 1). This resulted in a concurrent prevalence rate of 1.03%, indicating that approximately 1 in every 100 patients had both conditions.

### Comparative analysis of diabetes-related risk factors in adults with and without breast cancer

**Demographic characteristics**: Demographic analysis showed significant differences in gender, and body weight across study groups. Gender distribution showed a significant association with study groups (χ² = 108.65, *P* < 0.0001), with females being more prevalent in all groups, particularly in the Cancerous (100%) and Diabetic Cancerous (96%) groups, highlighting their higher vulnerability to these health conditions. Males were underrepresented, only 13% in the Diabetic group and 4% in the Diabetic Cancerous group (Table 3). Body weight distribution was significantly different between groups (F = 3.918, *P* < 0.009) (Table 4), with weights ranging from 38 kg to 110 kg. Notable weight peaks were observed at 70 kg (8%) and 80 kg (7%) in the Normal group, 55 kg (13%) and 80 kg (8%) in the Diabetic group, 50 kg (17%) and 70 kg (11%) in the Cancerous group, and 70 kg (14%) and 80 kg (9%) in the Diabetic Cancerous group.

**Table 3:** Relationship between study groups and gender distribution

Parameter	Groups					Chi-square (*P*-value)
	Normal (%) *n*=100	Diabetic (%) *n*=100	Cancerous (%) *n*=100	Diabetic Cancerous (%) *n*=100	Total (%) *n*=400	
*Gender*						
Male	49 (49%)	13 (13%)	0 (0%)	4 (4%)	66 (16.5%)	108.65^**^ *P*<0.0001
Female	51 (51%)	87 (87%)	100 (100%)	96 (96%)	334 (83.5%)	

NS = Non-significant (*P*>0.05); * = Significant (*P*<0.05); ** = Highly significant (*P*<0.01)

**Table 4:** Comparison of weight distribution across study groups using ANOVA

Parameters	Sum of Squares	df	Mean Square	F	Sig.
Weight	2996.010	3	998.670	3.918	0.009^**^

NS = Non-significant (*P*>0.05); * = Significant (*P*<0.05); ** = Highly significant (*P*<0.01)

**Clinical and medical history**: Clinical and medical history factors showed significant differences across health conditions. BMI distribution showed a significant association with the study groups (χ² = 20.68, *P* < 0.014), with obesity being the highest in the Diabetic group (41%), followed by Cancerous and Diabetic Cancerous groups both had 40% and Normal group had 23%. Fat accumulation was mainly in the abdomen in all groups (χ² = 103.21, *P* < 0.0001), peaking at 44% in the Normal group and decreasing in the Diabetic (32%), Diabetic Cancerous (31%), and Cancerous (30%) groups. Metabolic syndrome prevalence varied significantly (χ² = 26.71, *P* < 0.0001), with the highest comorbidity in the Diabetic group (72%) compared to the Normal (39%), Cancerous (49%), and Diabetic Cancerous (64%) groups (Table 5).

**Table 5:** Association of clinical and medical history with different health status groups among respondents

Parameters	Groups					Chi-square (*P*-value)
	Normal (%) *n*=100	Diabetic (%) *n*=100	Cancerous (%)* n*=100	Diabetic Cancerous (%)* n*=100	Total (%) *n*=400	
*BMI*						
Underweight	4 (4%)	0 (0%)	5 (5%)	1 (1%)	10 (2.5%)	20.68^*^ *P*<0.014
Normal weight	36 (36%)	30 (30%)	37 (37%)	31 (31%)	134 (33.5%)	
Overweight	37 (37%)	29 (29%)	18 (18%)	28 (28%)	112 (28%)	
Obese	23 (23%)	41 (41%)	40 (40%)	40 (40%)	144 (36%)	
*Fat Accumulation Areas*						
Abdomen	44 (44%)	32 (32%)	30 (30%)	31 (31%)	137 (34.2%)	103.21** *P*<0.0001
Buttocks	1 (1%)	2 (2%)	2 (2%)	6 (6%)	11 (2.8%)	
Thighs	7 (7%)	4 (4%)	0 (0%)	4 (4%)	15 (3.8%)	
Shoulders	0 (0%)	0 (0%)	0 (0%)	1 (1%)	1 (0.2%)	
Abdomen, Breast	0 (0%)	3 (3%)	0 (0%)	0 (0%)	3 (0.8%)	
Abdomen, Buttocks	1 (1%)	6 (6%)	2 (2%)	1 (1%)	10 (2.5%)	
Abdomin, Shoulders	0 (0%)	3 (3%)	0 (0%)	0 (0%)	3 (0.8%)	
Abdomin, Thighs	9 (9%)	16 (16%)	13 (13%)	20 (20%)	58 (14.5%)	
Thighs, Buttocks	5 (5%)	2 (2%)	2 (2%)	3 (3%)	12 (3%)	
Abdomen, Buttocks, Shoulders	1 (1%)	0 (0%)	0 (0%)	0 (0%)	1 (0.2%)	
Abdomen, Thigh, Breast	0 (0%)	3 (3%)	0 (0%)	0 (0%)	3 (0.8%)	
Abdomen, Thighs, Buttocks	4 (4%)	4 (4%)	18 (18%)	8 (8%)	34 (8.5%)	
Abdomin, Thighs, Shoulders	0 (0%)	2 (2%)	0 (0%)	0 (0%)	2 (0.5%)	
Abdomen, Breast, Thighs, Buttocks	0 (0%)	1 (1%)	0 (0%)	0 (0%)	1 (0.2%)	
Abdomen, Thighs, Buttocks, Breast	0 (0%)	2 (2%)	1 (1%)	0 (0%)	3 (0.8%)	
Abdomen, Thighs, Buttocks, Shoulders	0 (0%)	0 (%)	0 (0%)	3 (3%)	3 (0.8%)	
No	28 (28%)	20 (20%)	32 (32%)	23 (23%)	103 (25.8%)	
*Metabolic syndrome*						
No	61 (61%)	28 (28%)	51 (51%)	36 (36%)	176 (44%)	26.71^**^ *P* **<0.0001**
Yes	39 (39%)	72 (72%)	49 (49%)	64 (64%)	224 (56%)	

NS = Non-significant (*P*>0.05); * = Significant (*P*<0.05); ** = Highly significant (*P*<0.01)

**Dietary habits**: Fried fish consumption was highest in the Diabetic group (11%), followed by the Normal group (10%), while the Cancerous and Diabetic Cancerous groups had the lowest consumption rates (5% each). Occasional consumption was highest in the Normal group (83%), and lowest in the Diabetic group (66%). A significant association was observed between fried fish intake and health status (χ² = 32.99, *P* < 0.001). For red meat, the Normal group had the highest weekly consumption (25%), while the Diabetic Cancerous group had the lowest (14%). Conversely, occasional consumption was highest in the Diabetic Cancerous group (72%). These differences were significant association was found (χ² = 32.71, *P* < 0.0001). Processed meat consumption was highest in the Normal group (18%), with the Diabetic Cancerous group reporting the lowest weekly consumption (3%). A highly significant association was noted between processed meat consumption and health status (χ² = 43.20, *P* < 0.0001). Meat or poultry was most commonly consumed weekly by the Normal group (63%), and the Diabetic group had the lowest weekly consumption (45%). Statistical analysis revealed a significant association (χ² = 30.75, *P* < 0.0001). Egg consumption was highest in the Diabetic Cancerous group (58%), with a significant association identified (χ² = 22.54, *P* < 0.007). Fast food consumption was most common in the Normal group (15%), while the Cancerous group had the lowest weekly consumption (5%). A significant association was observed (χ² = 25.80, *P* < 0.002). Instant noodle consumption was highest in the Diabetic group (7%), with a significant association found (χ² = 29.84, *P* < 0.0001). Pickle consumption was highest in the Diabetic group (44%), and a significant association was noted (χ² = 29.77, *P* < 0.0001). Bakery products were most consumed by the Cancerous group (57%), but no significant association with health status was identified (χ² = 16.42, *p* > 0.059). For white bread, weekly consumption was highest in the Cancerous group (32%), with occasional consumption most prevalent in the Normal group (56%). Statistical analysis showed no significant association (χ² = 12.94, *P* > 0.165).

Regarding homemade butter, the Normal group reported the highest weekly consumption (33%), while the Cancerous group had the most individuals who used to consume it (25%), with no significant association found (χ² = 15.48, *P* > 0.079). Blue Band butter showed minimal consumption, with 1% of participants in the Normal and Diabetic Cancerous groups reporting weekly use, with no significant association (χ² = 11.58, *P* > 0.238). Whole milk consumption was highest in the Normal group (30% weekly), but non-consumption was most common in the Cancerous group (66%), with no significant association (χ² = 13.99, *P* > 0.123). Coffee consumption was lower overall, with no significant association found (χ² = 6.44, *P* > 0.695). Tea consumption was consistently high across all groups, but statistical analysis showed no significant association (χ² = 6.92, *P* > 0.646). Tea whitener consumption was also low, with the highest percentage of non-consumers in the Cancerous group (64%), and no significant association (χ² = 9.40, *P* > 0.401). Soft drink consumption varied, with the highest weekly consumption in the Diabetic Cancerous group (24%), and a significant association was found (χ² = 19.15, *P* < 0.024). Processed juice consumption was highest in the Diabetic and Normal groups (9% weekly), and a significant association was found (χ² = 27.98, *P* < 0.001). Citrus fruit juice was commonly consumed weekly by the Cancerous group (23%), and a significant association was found (χ² = 25.70, *P* < 0.002).

Fruit consumption was higher across all groups, with no significant association (χ² = 3.61, *P* > 0.306). Root vegetables were consumed weekly by almost all participants, with no significant association (χ² = 6.71, *P* > 0.349). For leafy vegetables (gobi, palak, salad), weekly intake was highest in the Cancerous group (80%), while the lowest intake was 75% in the Diabetic Cancerous group, with no significant differences (χ² = 2.667, *P* > 0.446). Lentil consumption was highest in the Normal group (70%) and lowest in the Diabetic Cancerous group (65%), with no significant differences (χ² = 0.814, *P* > 0.846). White rice consumption was highest in the Normal group (67%) and lowest in the Cancerous group (61%), showing no significant difference (χ² = 3.91, *P* > 0.689). White flour consumption was significantly different, with 70% of the Normal group consuming it occasionally, compared with 52% of the Cancerous group (χ² = 43.68, *P* < 0.0001). Nuts were consumed weekly by 27% of the Normal and Diabetic Cancerous groups, with no significant differences (χ² = 12.22, *P* > 0.201). White sugar intake was highest in the Cancerous group (84%) and lowest in the Diabetic Cancerous group (38%), with a highly significant difference (χ² = 86.47, *P* < 0.0001). Ice cream was consumed weekly by 10% of the Normal group and 2% of the Diabetic Cancerous group, with a significant difference (χ² = 26.20, *P* < 0.002). Chocolate consumption showed no significant difference (χ² = 14.05, *P* > 0.121). Deep-fried food consumption was highest in the Normal group (31%) and lowest in the Diabetic group (4%), with a highly significant difference (χ² = 52.38, *P* < 0.0001). Salt intake was relatively consistent across all groups, with no significant difference (χ² = 2.23, *P* > 0.526). The majority of participants in all groups reported avoiding fat while eating meat, with no significant difference (χ² = 4.556, *P* > 0.602). Finally, all groups reported not adhering to a specific diet (Tables 6 and 7).

**Table 6:** Association of dietary habits with different health status groups among respondents

Parameters	Groups					Chi-square (*P*-value)
	Normal (%) *n*=100	Diabetic (%) *n*=100	Cancerous (%) *n*=100	Diabetic Cancerous (%) *n*=100	Total (%) *n*=400	
*Fried Fish*						
Weekly	10 (10%)	11 (11%)	5 (5%)	5 (5%)	31 (7.8%)	32.99^**^ *P*<0.0001
Occasionally	83 (83%)	66 (66%)	67 (67%)	81 (81%)	297 (74.2%)	
Used to	5 (5%)	14 (14%)	7 (7%)	4 (4%)	30 (7.5%)	
Never	2 (2%)	9 (9%)	21 (21%)	10 (10%)	42 (10.5%)	
*Red meat*						
Weekly	25 (25%)	18 (18%)	16 (16%)	14 (14%)	73 (18.2%)	32.71^**^ *P*<0.0001
Occasionally	66 (66%)	60 (60%)	55 (55%)	72 (72%)	253 (63.2%)	
Used to	4 (4%)	16 (16%)	8 (8%)	6 (6%)	34 (8.5%)	
Never	5 (5%)	6 (6%)	21 (21%)	8 (8%)	40 (10%)	
*Processed meat*						
Weekly	18 (18%)	12 (12%)	4 (4%)	3 (3%)	37 (9.2%)	43.20^**^ *P*<0.0001
Occasionally	63 (63%)	53 (53%)	42 (42%)	63 (63%)	221 (55.2%)	
Used to	2 (2%)	9 (9%)	8 (8%)	3 (3%)	22 (5.5%)	
Never	17 (17%)	26 (26%)	46 (46%)	31 (31%)	120 (30%)	
*Meat or poultry*						
Weekly	63 (63%)	45 (45%)	61 (61%)	56 (56%)	225 (56.2%)	30.75^**^ *P*<0.0001
Occasionally	34 (34%)	40 (40%)	27 (27%)	42 (42%)	143 (35.8%)	
Used to	1 (1%)	12 (12%)	5 (5%)	2 (2%)	20 (5%)	
Never	2 (2%)	3 (3%)	7 (7%)	0 (0%)	12 (3%)	
*Egg*						
Weekly	57 (57%)	40 (40%)	54 (54%)	58 (58%)	209 (52.2%)	22.54^**^ *P*<0.007
Occasionally	33 (33%)	38 (38%)	38 (38%)	35 (35%)	144 (36%)	
Used to	10 (10%)	17 (17%)	8 (8%)	6 (6%)	41 (10.2%)	
Never	0 (0%)	5 (5%)	0 (0%)	1 (1%)	6 (1.5%)	
*Fast food (pizza, burger, fries etc.)*						
Weekly	15 (15%)	8 (8%)	5 (5%)	6 (6%)	34 (8.5%)	25.80^**^ *P*<0.002
Occasionally	54 (54%)	45 (45%)	38 (38%)	52 (52%)	189 (47.2%)	
Used to	3 (3%)	13 (13%)	14 (14%)	4 (4%)	34 (8.5%)	
Never	28 (28%)	34 (34%)	43 (43%)	38 (38%)	143 (35.8%)	
*Instant noodles*						
Weekly	1 (1%)	7 (7%)	2 (2%)	1 (1%)	11 (2.8%)	29.84^**^ *P*<0.0001
Occasionally	24 (24%)	34 (34%)	23 (23%)	25 (25%)	106 (26.5%)	
Used to	3 (3%)	12 (12%)	5 (5%)	2 (2%)	22 (5.5%)	
Never	72 (72%)	47 (47%)	70 (70%)	72 (72%)	261 (65.2%)	
*Pickle*						
Weekly	41 (41%)	44 (44%)	40 (40%)	28 (28%)	153 (38.2%)	29.77^**^ *P*<0.0001
Occasionally	45 (45%)	38 (38%)	29 (29%)	47 (47%)	159 (39.8%)	
Used to	10 (10%)	17 (17%)	19 (19%)	23 (23%)	69 (17.2%)	
Never	4 (4%)	1 (1%)	12 (12%)	2 (2%)	19 (4.8%)	
*Bakery products (Rusk, cake rusk, etc.)*						
Weekly	55 (55%)	38 (38%)	57 (57%)	52 (52%)	202 (50.5%)	16.42^NS^ *P***>**0.059
Occasionally	45 (45%)	55 (55%)	36 (36%)	44 (44%)	180 (45%)	
Used to	0 (0%)	4 (4%)	5 (5%)	3 (3%)	12 (3%)	
Never	0 (0%)	3 (3%)	2 (2%)	1 (1%)	6 (1.5%)	
*White bread*						
Weekly	21 (21%)	24 (24%)	32 (32%)	29 (29%)	106 (26.5%)	12.94^NS^ *P***>**0.165
Occasionally	56 (56%)	53 (53%)	45 (45%)	53 (53%)	207 (51.8%)	
Used to	3 (3%)	6 (6%)	10 (10%)	2 (2%)	21 (5.2%)	
Never	20 (20%)	17 (17%)	13 (13%)	16 (16%)	66 (16.5%)	
*Homemade butter*						
Weekly	33 (33%)	25 (25%)	18 (18%)	18 (18%)	94 (23.5%)	15.48^NS^ *P***>**0.079
Occasionally	27 (27%)	27 (27%)	24 (24%)	36 (36%)	114 (28.5%)	
Used to	21 (21%)	27 (27%)	25 (25%)	20 (20%)	93 (23.2%)	
Never	19 (19%)	21 (21%)	33 (33%)	26 (26%)	99 (24.8%)	
*Blue band butter*						
Weekly	1 (1%)	0 (0%)	0 (0%)	1 (1%)	2 (0.5%)	11.58^NS^ *P***>**0.238
Occasionally	6 (6%)	10 (10%)	5 (5%)	1 (1%)	22 (5.5%)	
Used to	7 (7%)	4 (4%)	7 (7%)	4 (4%)	22 (5.5%)	
Never	86 (86%)	86 (86%)	88 (88%)	94 (94%)	354 (88.5%)	
*Whole milk*						
Weekly	30 (30%)	28 (28%)	28 (28%)	21 (21%)	107 (26.8%)	13.99^NS^ *P***>**0.123
Occasionally	6 (6%)	5 (5%)	3 (3%)	3 (3%)	17 (4.2%)	
Used to	2 (2%)	9 (9%)	3 (3%)	2 (2%)	16 (4%)	
Never	62 (62%)	58 (58%)	66 (66%)	74 (74%)	260 (65%)	
*Coffee*						
Weekly	10 (10%)	5 (5%)	4 (4%)	5 (5%)	24 (6%)	6.44^NS^ *P*>0.695
Occasionally	15 (15%)	15 (15%)	14 (14%)	16 (16%)	60 (15%)	
Used to	5 (5%)	10 (10%)	6 (6%)	9 (9%)	30 (7.5%)	
Never	70 (70%)	70 (70%)	76 (76%)	70 (70%)	286 (71.5%)	
*Tea*						
Weekly	88 (88%)	92 (92%)	92 (92%)	91 (91%)	363 (90.8%)	6.92^NS^ *P***>**0.646
Occasionally	9 (9%)	8 (8%)	6 (6%)	6 (6%)	29 (7.2%)	
Used to	2 (2%)	0 (0%)	2 (2%)	1 (1%)	5 (1.2%)	
Never	1 (1%)	0 (0%)	0 (0%)	2 (2%)	3 (0.8%)	
*Tea whitener*						
Weekly	3 (3%)	3 (3%)	2 (2%)	2 (2%)	10 (2.5%)	9.40^NS^ *P***>**0.401
Occasionally	16 (16%)	21 (21%)	8 (8%)	16 (16%)	61 (15.2%)	
Used to	24 (24%)	20 (20%)	26 (26%)	17 (17%)	87 (21.8%)	
Never	57 (57%)	56 (56%)	64 (64%)	65 (65%)	242 (60.5%)	
*Soft drinks*						
Weekly	30 (30%)	13 (13%)	19 (19%)	24 (24%)	86 (21.5%)	19.15^*^ *P***<**0.024
Occasionally	60 (60%)	57 (57%)	62 (62%)	56 (56%)	235 (58.8%)	
Used to	8 (8%)	22 (22%)	16 (16%)	14 (14%)	60 (15%)	
Never	2 (2%)	8 (8%)	3 (3%)	6 (6%)	19 (4.8%)	
*Processed fruit-juice*						
Weekly	9 (9%)	9 (9%)	8 (8%)	5 (5%)	31 (7.8%)	27.98^**^ *P***<**0.001
Occasionally	54 (54%)	49 (49%)	65 (65%)	49 (49%)	217 (54.2%)	
Used to	8 (8%)	21 (21%)	17 (17%)	10 (10%)	56 (14%)	
Never	29 (29%)	21 (21%)	10 (10%)	36 (36%)	96 (24%)	
*Citrus fruit juices*						
Weekly	19 (19%)	9 (9%)	23 (23%)	11 (11%)	62 (15.5%)	25.70^**^ *P***<**0.002
Occasionally	50 (50%)	36 (36%)	48 (48%)	43 (43%)	177 (44.2%)	
Used to	2 (2%)	11 (11%)	5 (5%)	6 (6%)	24 (6%)	
Never	29 (29%)	44 (44%)	24 (24%)	40 (40%)	137 (34.2%)	
*Fruits*						
Weekly	76 (76%)	78 (78%)	85 (85%)	75 (75%)	314 (78.5%)	3.6^NS^ *P*>0.306
Occasionally	24 (24%)	22 (22%)	15 (15%)	25 (25%)	86 (21.5%)	
Used to	0 (0%)	0 (0%)	0 (0%)	0 (0%)	0 (0%)	
Never	0 (0%)	0 (0%)	0 (0%)	0 (0%)	0 (0%)	
*Root vegetables (shljm, chukndr, adrk, alo)*						
Weekly	100 (100%)	98 (98%)	100 (100%)	98 (98%)	396 (99%)	6.7^NS^ *P*>0.349
Occasionally	0 (0%)	2 (2%)	0 (0%)	1 (1%)	3 (0.8%)	
Used to	0 (0%)	0 (0%)	0 (0%)	1 (1%)	1 (0.2%)	
Never	0 (0%)	0 (0%)	0 (0%)	0 (0%)	0 (0%)	
*Leafy vegetables (gobi, plk, salad ky pty)*						
Weekly	75 (75%)	70 (70%)	80 (80%)	75 (75%)	300 (75%)	2.667^NS^ *P*>0.446
Occasionally	25 (25%)	30 (30%)	20 (15%)	25 (25%)	100 (25%)	
Used to	0 (0%)	0 (0%)	0 (0%)	0 (0%)	0 (0%)	
Never	0 (0%)	0 (0%)	0 (0%)	0 (0%)	0 (0%)	
*Lentils*						
Weekly	70 (70%)	65 (65%)	68 (68%)	65 (65%)	268 (67%)	0.814^NS^ *P*>0.846
Occasionally	30 (30%)	35 (35%)	32 (32%)	35 (35%)	132 (33%)	
Used to	0 (0%)	0 (0%)	0 (0%)	0 (0%)	0 (0%)	
Never	0 (0%)	0 (0%)	0 (0%)	0 (0%)	0 (0%)	
*White rice*						
Weekly	67 (67%)	62 (62%)	61 (61%)	66 (66%)	256 (64%)	3.9^NS^ *P***>**0.689
Occasionally	29 (29%)	28 (28%)	33 (33%)	28 (28%)	118 (29.5%)	
Used to	4 (4%)	10 (10%)	6 (6%)	6 (6%)	26 (6.5%)	
Never	0 (0%)	0 (0%)	0 (0%)	0 (0%)	0 (0%)	
*White flour*						
Weekly	15 (15%)	7 (7%)	6 (6%)	5 (5%)	33 (8.2%)	43.68^**^ *P* **<0.0001**
Occasionally	70 (70%)	83 (83%)	52 (52%)	75 (75%)	280 (70%)	
Used to	3 (3%)	2 (2%)	10 (10%)	7 (7%)	22 (5.5%)	
Never	12 (12%)	8 (8%)	32 (32%)	13 (13%)	65 (16.2%)	
*Nuts*						
Weekly	27 (27%)	19 (19%)	17 (17%)	27 (27%)	90 (22.5%)	12.22^NS^ *P***>**0.201
Occasionally	63 (63%)	63 (63%)	75 (75%)	59 (59%)	260 (65%)	
Used to	4 (4%)	10 (10%)	6 (6%)	8 (8%)	28 (7%)	
Never	6 (6%)	8 (8%)	2 (2%)	6 (6%)	22 (5.5%)	
*White sugar*						
Weekly	82 (82%)	52 (52%)	84 (84%)	38 (38%)	256 (64%)	86.47^**^ *P*<0.0001
Occasionally	17 (17%)	34 (34%)	10 (10%)	57 (57%)	118 (29.5%)	
Used to	1 (1%)	14 (14%)	6 (6%)	4 (4%)	25 (6.2%)	
Never	0 (0%)	0 (0%)	0 (0%)	1 (1%)	1 (0.2%)	
*Ice cream*						
Weekly	10 (10%)	3 (3%)	11 (11%)	2 (2%)	26 (6.5%)	26.20^**^ *P*<0.002
Occasionally	73 (73%)	78 (78%)	58 (58%)	71 (71%)	280 (70%)	
Used to	8 (8%)	15 (15%)	12 (12%)	14 (14%)	49 (12.2%)	
Never	9 (9%)	4 (4%)	19 (19%)	13 (13%)	45 (11.2%)	
*Chocolate*						
Weekly	12 (12%)	7 (7%)	8 (8%)	3 (3%)	30 (7.5%)	14.05^NS^ *P*>0.121
Occasionally	39 (39%)	39 (39%)	42 (42%)	48 (48%)	168 (42%)	
Used to	13 (13%)	24 (24%)	18 (18%)	11 (11%)	66 (16.5%)	
Never	36 (36%)	30 (30%)	32 (32%)	38 (38%)	136 (34%)	
*Deep-fried food items*						
Weekly	31 (31%)	4 (4%)	9 (9%)	10 (10%)	54 (13.5%)	52.38^**^ *P* **<0.0001**
Occasionally	63 (63%)	95 (95%)	79 (79%)	78 (78%)	315 (78.8%)	
Used to	2 (2%)	1 (1%)	7 (7%)	9 (9%)	19 (4.8%)	
Never	4 (4%)	0 (0%)	5 (5%)	3 (3%)	12 (3%)	
*Salt intake*						
1-2 spoons	99 (99%)	100 (100%)	98 (98%)	98 (98%)	395 (98.8%)	2.23^NS^ *P***>**0.526
3-4 spoons	1 (1%)	0 (0%)	2 (2%)	2 (2%)	5 (1.2%)	
5-6 spoons	0 (0%)	0 (0%)	0 (0%)	0 (0%)	0 (0%)	
*Avoid fat while eating meat*						
Yes	72 (72%)	80 (80%)	75 (75%)	80 (80%)	307 (76.8%)	4.556^NS^ *P***>**0.602
No	21 (21%)	18 (18%)	21 (21%)	16 (16%)	76 (19%)	
Sometimes	7 (7%)	2 (2%)	4 (4%)	4 (4%)	17 (4.2%)	
*Follow any specific diet*						
No	100 (100%)	100 (100%)	100 (100%)	100 (100%)	400 (100%)	**-**
Mediterranean diet	0 (0%)	0 (0%)	0 (0%)	0 (0%)	0 (0%)	
Vegetarian diet	0 (0%)	0 (0%)	0 (0%)	0 (0%)	0 (0%)	
Vegan diet	0 (0%)	0 (0%)	0 (0%)	0 (0%)	0 (0%)	
Ketogenic diet	0 (0%)	0 (0%)	0 (0%)	0 (0%)	0 (0%)	
Non-vegetarian	0 (0%)	0 (0%)	0 (0%)	0 (0%)	0 (0%)	
Other	0 (0%)	0 (0%)	0 (0%)	0 (0%)	0 (0%)	

NS = Non-significant (*P*>0.05); * = Significant (*P*<0.05); ** = Highly significant (*P*<0.01)

**Table 7:** Descriptive statistics of respondents’ eating habits and cooking practices across study groups: frequency of food item consumption

Food Items	Groups											
	Normal			Diabetic			Cancerous			Diabetic Cancerous		
	*N*	Mean	SE	*N*	Mean	SE	*N*	Mean	SE	*N*	Mean	SE
Fish	10	1.70	0.40	11	2.09	0.39	5	1.80	0.37	5	1.60	0.24
Red meat	25	1.52	0.15	18	1.39	0.20	16	1.62	0.20	14	1.79	0.42
Processed meat	18	1.83	0.35	12	1.42	0.19	4	1.00	0.00	3	2.00	0.58
Meat or poultry	63	2.13	0.19	45	2.49	0.26	61	1.92	0.19	56	1.71	0.14
Eggs	57	3.68	0.31	40	3.17	0.32	54	3.54	0.31	58	3.48	0.32
Fast food	15	1.80	0.28	8	1.63	0.26	5	1.00	0.00	6	1.17	0.17
Instant noodles	1	1.00	.	7	1.57	0.30	2	1.00	0.00	1	1.00	.
Pickles	41	3.02	0.33	44	3.09	0.30	40	3.03	0.36	28	3.54	0.48
Bakery products	55	3.33	0.31	38	4.26	0.37	57	3.32	0.29	52	3.63	0.33
White bread	21	2.38	0.41	24	3.42	0.54	32	2.34	0.30	29	2.00	0.34
Homemade butter	33	5.21	0.43	24	5.50	0.49	18	4.89	0.60	18	5.94	0.48
Blueband butter	1	1.00	.	0	.	.	0	.	.	1	3.00	.
Drink whole milk	30	6.87	0.13	28	6.79	0.21	28	6.64	0.25	21	6.76	0.24
Drink coffee	10	5.20	2.13	1	1.00	.	0	.	.	1	3.00	.
Drink tea	88	15.00	0.71	92	14.63	0.73	92	15.52	0.74	91	13.38	0.63
Tea whitener	3	7.00	0.00	3	2.00	0.00	2	2.00	0.00	2	5.00	2.00
Soft drinks	30	3.47	0.43	13	2.00	0.45	19	1.79	0.21	24	2.83	0.38
Processed fruit juices	9	2.00	0.33	9	2.11	0.26	8	1.13	0.12	5	3.00	1.00
Citrus fruit juices	19	2.47	0.39	9	1.89	0.31	23	3.83	0.54	11	4.00	0.76
Fruits	76	4.34	0.27	78	3.94	0.27	85	4.86	0.27	75	5.05	0.28
Root vegetables	100	4.09	0.16	98	4.49	0.16	100	3.89	0.15	98	4.04	0.13
Leafy vegetables	83	1.66	0.10	59	1.71	0.16	85	1.96	0.12	75	1.76	0.12
Lentils	89	1.76	0.10	77	2.32	0.14	94	1.59	0.09	86	1.56	0.08
White rice	67	2.15	0.15	62	1.95	0.15	61	1.72	0.10	66	2.02	0.15
White flour	15	2.00	0.54	7	3.14	0.34	6	1.67	0.33	5	4.60	1.47
Nuts	27	3.89	0.49	19	4.05	0.54	17	3.41	0.68	27	5.04	0.48
White sugar	82	7.00	0.00	53	7.00	0.00	84	7.00	0.00	38	6.92	0.08
Ice cream	10	1.20	0.13	3	2.00	0.00	11	3.64	0.82	2	1.50	0.50
Chocolate	12	3.08	0.61	7	1.86	0.86	8	4.13	1.09	3	3.33	1.86
Deep-fried items	31	2.10	0.29	4	1.00	0.00	9	1.00	0.00	10	1.20	0.13
Artificial food color	12	1.83	0.51	3	2.00	0.00	9	1.00	0.00	6	1.17	0.17
Aluminum foil	2	1.50	0.50	0	.	.	0	.	.	0	.	.

**Kitchen practices**: Kitchen practices varied across groups but generally lacked significant associations with health status. Food color consumption was predominantly occasional across all groups, with no significant association (χ² = 15.74, *P* > 0.073). Aluminum foil and utensil usage were minimal, with most participants never using them, although aluminum foil showed a borderline significant trend (χ² = 16.62, *P* < 0.055). Ghee usage was highest in the Cancerous and Diabetic Cancerous groups (57%), while oil use also peaked in the Normal group (49%), no significant association was observed (χ² = 10.16, *P* > 0.118). Overcooking oil was significantly associated with health status (χ² = 25.17, *P* < 0.003), most common in the Diabetic group (89%). Reuse of cooked oil and microwave usage showed consistent patterns across groups, with no significant differences observed (*P* > 0.05) (Table 8).

**Table 8:** Association of kitchen practices with different health status groups among respondents

Kitchen Practices	Groups					Chi-square (*P*-value)
	Normal (%) *n*=100	Diabetic (%) *n*=100	Cancerous (%) *n*=100	Diabetic Cancerous (%) *n*=100	Total (%) *n*=400	
*Food color*						
Weekly	12 (12%)	3 (3%)	9 (9%)	6 (6%)	30 (7.5%)	15.74^NS^ *P*>0.073
Occasionally	70 (70%)	77 (77%)	63 (63%)	70 (70%)	280 (70%)	
Used to	3 (3%)	10 (10%)	8 (8%)	5 (5%)	26 (6.5%)	
Never	15 (15%)	10 (10%)	20 (20%)	19 (19%)	64 (16%)	
*Aluminum foils are used for cooking/baking/storing*						
Weekly	2 (2%)	0 (0%)	0 (0%)	2 (2%)	4 (1%)	16.62^*^ *P***<**0.055
Occasionally	5 (5%)	6 (6%)	0 (0%)	2 (2%)	13 (3.2%)	
Used to	2 (2%)	0 (0%)	3 (3%)	0 (0%)	5 (1.2%)	
Never	91 (91%)	94 (94%)	97 (97%)	96 (96%)	378 94.5(%)	
*Aluminum utensils are used for cooking*						
Never	90 (90%)	97 (97%)	94 (94%)	97 (97%)	378 (94.5%)	9.95^NS^ *P***>**0.127
Regularly	10 (10%)	3 (3%)	6 (6%)	3 (3%)	20 (5%)	
Used to	0 (0%)	0 (0%)	0 (0%)	0 (0%)	2 (0.5%)	
*Ghee/oil usage for cooking*						
Ghee	41(%)	52(%)	57(%)	57(%)	207(%)	10.16^NS^ *P***>**0.118
Oil	49(%)	33(%)	33(%)	33(%)	148(%)	
Both	10(%)	15(%)	10(%)	10(%)	45(%)	
*Overcooked oil*						
Never	19 (19%)	11 (11%)	33 (33%)	20 (20%)	83 (20.8%)	25.17^**^ *P***<**0.003
Occasionally	0 (0%)	0 (0%)	3 (3%)	1 (1%)	4 (1%)	
Regularly	81 (%)	89 (89%)	64 (64%)	78 (78%)	312 (78%)	
Used to	0 (0%)	0 (0%)	0 (0%)	1 (1%)	1 (0.2%)	
*Repeatedly use the same cooked oil for cooking/frying*						
Never	23 (23%)	19 (19%)	16 (16%)	14 (14%)	72 (18%)	9.48^NS^ *P***>**0.148
Occasionally	77 (77%)	79 (79%)	81 (81%)	80 (80%)	317 (79.2%)	
Regularly	0 (0%)	2 (2%)	3 (3%)	6 (6%)	11 (2.8%)	
*Microwave usage*						
Never	49 (49%)	57 (57%)	59 (59%)	52 (52%)	217 (54.2%)	8.51^NS^ *P***>**0.484
Used to	6 (6%)	5 (5%)	6 (6%)	5 (5%)	22 (5.5%)	
Occasionally	14 (14%)	21 (21%)	16 (16%)	16 (16%)	67 (16.8%)	
Regularly	31 (31%)	17 (17%)	19 (19%)	27 (27%)	94 (23.5%)	

NS = Non-significant (*P*>0.05); * = Significant (*P*<0.05); ** = Highly significant (*P*<0.01)

**Behavioral factors**: Physical activity patterns differed significantly across groups, with sedentary lifestyles most prevalent in Diabetic Cancerous (88%) and Cancerous (85%) groups, compared to the Normal group (60%), demonstrating a strong association (χ² = 49.47, *P* < 0.0001). Sleep duration was also significantly different, with Cancerous participants showing significantly shorter sleep rates (35% for 3–4 h) than the other groups (χ² = 23.77, *P* < 0.001). Screen time patterns indicated ≤ 2 h of usage in the Diabetic group (88%), with a strong association to health status (χ² = 34.94, *P* < 0.0001). Stress levels were notably higher in the Diabetic group (45% moderate stress) compared to others (χ² = 29.47, *P* < 0.0001). Hair dye and deodorant usage showed significant associations with health conditions (χ² = 105.04, *P* < 0.0001; χ² = 25.24, *P* < 0.003, respectively). Conversely, facewash, supplements, smoking, drug use, vaping, chewing tobacco/snuff, and alcohol consumption exhibited no significant associations with health conditions (Table 9).

**Table 9:** Association of behavioral habits with different health status groups among respondents

Behavioral Habits	Groups					Chi-square (*P*-value)
	Normal (%) *n*=100	Diabetic (%) *n*=100	Cancerous (%) *n*=100	Diabetic Cancerous (%) *n*=100	Total (%) *n*=400	
*Physical activity*						
Sedentary	60 (60%)	69 (69%)	85 (85%)	88 (88%)	302 (75.5%)	49.47^**^ *P* **<0.0001**
Mild	28 (28%)	31 (31%)	13 (13%)	12 (12%)	84 (21%)	
Moderate	11 (11%)	0 (0%)	2 (2%)	0 (0%)	13 (3.2%)	
Extremely active	1 (1%)	0 (0%)	0 (0%)	0 (0%)	1 (0.2%)	
*Hours of sleep at night*						
3–4 h	16 (16%)	15 (15%)	35 (35%)	25 (25%)	91 (22.8%)	23.77^**^ *P***<**0.001
5–6 h	26 (26%)	28 (28%)	35 (35%)	30 (30%)	119 (29.8%)	
7–8 h	58 (58%)	57 (57%)	30 (30%)	45 (45%)	190 (47.5%)	
*Screen time*						
≤ 2 h	59 (59%)	88 (88%)	83 (83%)	79 (79%)	309 (77.2%)	34.94^**^ *P* **<0.0001**
3–5 h	23 (23%)	6 (6%)	12 (12%)	15 (15%)	56 (14%)	
6–8 h	9 (9%)	0 (0%)	3 (3%)	2 (2%)	14 (3.5%)	
≥ 9 h	9 (9%)	6 (6%)	2 (2%)	4 (4%)	21 (5.2%)	
*Stress level*						
Mild	68 (68%)	43 (43%)	65 (65%)	67 (67%)	243 (60.8%)	29.47^**^ *P***<0.0001**)
Moderate	15 (15%)	45 (45%)	25 (25%)	19 (19%)	104 (26%)	
Severe	17 (17%)	12 (12%)	10 (10%)	14 (14%)	53 (13.2%)	
*Hair dye usage*						
Never	50 (50%)	23 (23%)	28 (28%)	30 (30%)	131 (32.8%)	105.04^**^ *P* **<0.0001**
Used to dye	0 (0%)	5 (5%)	26 (26%)	42 (42%)	73 (18.2%)	
Occasionally	24 (24%)	49 (49%)	35 (35%)	21 (21%)	129 (32.2%)	
Monthly	26 (26%)	23 (23%)	11 (11%)	7 (7%)	67 (16.8%)	
*Facewash usage*						
Never	82 (82%)	82 (82%)	78 (78%)	87 (87%)	329 (82.2%)	10.85^NS^ *P***>**0.286
Used to wash	0 (0%)	0 (0%)	3 (3%)	2 (2%)	5 (1.2%)	
Occasionally	9 (9%)	13 (13%)	12 (12%)	8 (8%)	42 (10.5%)	
Regularly	9 (9%)	5 (5%)	7 (7%)	3 (3%)	24 (6%)	
*Deodorant usage*						
Never	92 (92%)	97 (97%)	92 (92%)	97 (97%)	378 (94.5%)	25.24^**^ *P***<**0.003
Used to	0 (0%)	0 (0%)	2 (2%)	1 (1%)	3 (0.8%)	
Occasionally	2 (2%)	3 (3%)	6 (6%)	2 (2%)	13 (3.2%)	
Regularly	6 (6%)	0 (0%)	0 (0%)	0 (0%)	6 (1.5%)	
*Supplements usage*						
Never	49 (49%)	44 (44%)	40 (40%)	30 (30%)	163 (40.8%)	10.801^NS^ *P*>0.290
Former	10 (10%)	10 (10%)	15 (15%)	12 (12%)	47 (11.8%)	
Occasionally	21 (21%)	18 (18%)	20 (20%)	25 (25%)	83 (21.0%)	
Regularly	20 (20%)	28 (28%)	25 (25%)	33 (33%)	106 (26.5%)	
*Smoking*						
Never	79 (79%)	96 (96%)	97 (97%)	96 (96%)	368 (92%)	40.11^**^ *P* **<0.0001**
Former	5 (5%)	0 (0%)	0 (0%)	0 (0%)	5 (1.2%)	
Occasionally	3 (3%)	0 (0%)	3 (3%)	1 (1%)	7 (1.8%)	
Regularly	13 (13%)	4 (4%)	0 (0%)	3 (3%)	20 (5%)	
*Drugs use*						
Never	98 (98%)	100 (100%)	100 (100%)	100 (100%)	398 (99.5%)	6.03^NS^ *P***>**0.420
Former	1 (1%)	0 (0%)	0 (0%)	0 (0%)	1 (0.2%)	
Occasionally	1 (1%)	0 (0%)	0 (0%)	0 (0%)	1 (0.2%)	
Regularly	0 (0%)	0 (0%)	0 (0%)	0 (0%)	0 (0%)	
*Vape*						
Never	98 (98%)	100 (100%)	100 (100%)	100 (100%)	398 (99.5%)	6.03^NS^ *P***>**0.110
Former	0 (0%)	0 (0%)	0 (0%)	0 (0%)	0 (0%)	
Occasionally	0 (0%)	0 (0%)	0 (0%)	0 (0%)	0 (0%)	
Regularly	2 (2%)	0 (0%)	0 (0%)	0 (0%)	2 (0.5%)	
*Chewing tobacco/Snuff*						
Never	96 (96%)	100 (100%)	100 (100%)	98 (98%)	394 (98.5%)	7.45^NS^ *P***>**0.282
Former	2 (2%)	0 (0%)	0 (0%)	1 (1%)	3 (0.8%)	
Occasionally	0 (0%)	0 (0%)	0 (0%)	0 (0%)	0 (0%)	
Regularly	2 (2%)	0 (0%)	0 (0%)	1 (1%)	3 (0.8%)	
*Alcohol consumption*						
Never	98 (98%)	100 (100%)	100 (100%)	100 (100%)	398 (99.5%)	6.03^NS^ *P***>**0.110
Former	2 (2%)	0 (0%)	0 (0%)	0 (0%)	2 (0.5%)	
Occasionally	0 (0%)	0 (0%)	0 (0%)	0 (0%)	0 (0%)	
Regularly	0 (0%)	0 (0%)	0 (0%)	0 (0%)	0 (0%)	

NS = Non-significant (*P*>0.05); * = Significant (*P*<0.05); ** = Highly significant (*P*<0.01)

**Environmental factors**: Exposure to occupational hazards did not significantly differ across health conditions, with 95% of the Normal group and 98% of Diabetic, Cancerous, and Diabetic Cancerous groups reporting no exposure (χ² = 14.94, *P* > 0.245). However, exposure to radiation and chemicals showed a significant association (χ² = 25.48, *P* < 0.013), with higher exposure in the Cancerous (4%) and Diabetic Cancerous (3%) groups compared to others. Regarding secondhand smoke, most participants in all groups reported never being exposed, and no significant association was found (χ² = 14.91, *P* > 0.094), indicating similar exposure levels across health conditions (Table 10).

**Table 10:** Association of environmental factors with different health status groups among respondents

Environmental Factors	Groups					Chi-square (*P*-value)
	Normal (%) *n*=100	Diabetic (%) *n*=100	Cancerous (%) *n*=100	Diabetic Cancerous (%) *n*=100	Total (%) *n*=400	
*Exposure to gases, stone dust, coal dust, wood dust, textile dust or fumes of any sort at work*						
Never	95 (95%)	98 (98%)	98 (98%)	98 (98%)	389 (97.2%)	14.94^NS^ *P***>**0.245
≤ 2 years	2 (2%)	0 (0%)	0 (0%)	0 (0%)	2 (0.5%)	
3-5 years	0 (0%)	0 (0%)	0 (0%)	1 (1%)	1 (0.2%)	
≥ 5 years	1 (1%)	0 (0%)	2 (2%)	0 (0%)	3 (0.8%)	
Currently	2 (2%)	2 (2%)	0 (0%)	1 (1%)	5 (1.2%)	
*Exposure to radiation, x-ray chemicals, solvents or oil products at work*						
Never	95 (95%)	100 (100%)	96 (96%)	97 (97%)	388 (97%)	25.48^*^ *P***<**0.013
≤ 2 years	0 (0%)	0 (0%)	0 (0%)	3 (3%)	3 (0.8%)	
3-5 years	1 (1%)	0 (0%)	0 (0%)	0 (0%)	1 (0.2%)	
≥ 5 years	2 (2%)	0 (0%)	0 (0%)	0 (0%)	2 (0.5%)	
Currently	2 (2%)	0 (0%)	4 (4%)	0 (0%)	6 (1.5%)	
*Exposure to secondhand smoke*						
Never	49 (49%)	59 (59%)	67 (67%)	64 (64%)	239 (59.8%)	14.91^NS^ *P***>**0.094
Used to exposed	6 (6%)	0 (0%)	5 (5%)	6 (6%)	17 (4.2%)	
Occasionally	24 (24%)	23 (23%)	14 (14%)	16 (16%)	77 (19.2%)	
Regularly	21 (21%)	18 (18%)	14 (14%)	14 (14%)	67 (16.8%)	

NS = Non-significant (*P*>0.05); * = Significant (*P*<0.05); ** = Highly significant (*P*<0.01)

**Female-Related factors**: Significant differences in menstruation status were observed across health conditions, with a higher percentage of postmenopausal participants in the Diabetic Cancerous group (89.6%) than in the other groups (χ² = 63.64, *P* < 0.0001). There was no significant association between health conditions and prevalence of PCOS (χ² = 2.34, *P* > 0.505). Oral contraceptive use showed significant variation (χ² = 8.79, *P* < 0.032), with higher usage in the Cancerous group (15%) and lower usage in the Diabetic group (2.3%). A significant association was found between previous history of gestational diabetes and current health conditions, with the Diabetic group showing the highest prevalence at 13.8% (χ² = 21.28, *P* < 0.0001). Makeup use was strongly associated with health conditions, with higher usage in the Cancerous (19%) and Diabetic Cancerous (37.5%) groups than others (χ² = 54.49, *P* < 0.0001) (Table 11).

**Table 11:** Association of female-specific characteristics with different health status groups among respondents

Parameters	Groups					Chi-square (*P*-value)
	Normal (%) *n*=87	Diabetic (%) *n*=100	Cancerous (%) *n*=96	Diabetic Cancerous (%) *n*=51	Total (%) *n*=334	
*Current menstruation status*						
Still menstruating	31 (60.8%)	44 (50.6%)	39 (39%)	8 (8.3%)	122 (36.5%)	63.64^**^ *P* **<0.0001**
In menopause	0 (0%)	5 (5.7%)	0 (0%)	2 (2.1%)	7 (2.1%)	
Post-menopause	20 (39.2%)	38 (43.7%)	61 (61%)	86 (89.6%)	205 (61.4%)	
*PCOS*						
Yes	7 (13.7%)	16 (18.4%)	11 (11%)	12 (12.5%)	46 (13.8%)	2.34^NS^ *P***>**0.505
No	44 (86.3%)	71 (81.6%)	89 (89%)	84 (87.5%)	288 (86.2%)	
*Ever taken oral contraceptives*						
Yes	6 (11.8%)	2 (2.3%)	15 (15%)	10 (10.4%)	33 (9.9%)	8.79^*^ *P***<**0.032
No	45 (88.2%)	85 (97.7%)	85 (85%)	86 (89.6%)	301 (90.1%)	
*Gestational diabetes*						
Yes	0 (0%)	12 (13.8%)	2 (2%)	2 (2.1%)	16 (4.8%)	21.28^**^ *P* **<0.0001**
No	51 (100%)	75 (86.2%)	98 (98%)	94 (97.9%)	318 (95.2%)	
*Makeup use*						
Never	18 (35.3%)	33 (37.9%)	29 (29%)	29 (30.2%)	109 (32.6%)	54.49^**^ *P* **<0.0001**
Used to	3 (5.9%)	12 (13.8%)	19 (19%)	36 (37.5%)	70 (21%)	
Occasionally	25 (49%)	42 (48.3%)	52 (52%)	31 (32.3%)	150 (44.9%)	
Regularly	5 (9.8%)	0 (0%)	0 (0%)	0 (0%)	5(1.5%)	

NS = Non-significant (*P*>0.05); * = Significant (*P*<0.05); ** = Highly significant (*P*<0.01)

### Recommendation for health care department

To address the dual burden of diabetes and breast cancer, a comprehensive, evidence-based approach is essential. Preventive strategies should be designed based on demographic and clinical characteristics to effectively manage high-risk individuals, with a strong focus on modifiable factors such as diet, physical activity, and environmental exposures. Personalized nutritional interventions, supported by advances in nutrigenomics, aim to reduce the risks associated with both conditions. Public health campaigns should focus on promoting healthy cooking practices, reducing consumption of harmful oil, and managing obesity. Additionally, mentorship programs linking healthcare providers experienced in diabetes and cancer management can enhance patient care, supporting both physical and mental health. The integration of wearable technology for continuous monitoring of glucose, BMI, and cancer biomarkers allows for real-time tracking and early intervention. AI-powered mobile applications can further improve adherence to treatment protocols by providing personalized health advice and reminders. Interdisciplinary collaboration through workshops involving endocrinologists, oncologists, dietitians, and mental health professionals can promote integrated care strategies. This approach, combining early detection, prevention, and individualized care, offers the potential to improve patient outcomes and establish a holistic framework for managing patients with diabetes and breast cancer.

## Discussion

Concurrent prevalence rate of 1.03%, this statistic indicates that approximately 1 in every 100 patients had both diabetes mellitus and breast cancer. This rate is consistent with European studies, where prevalence rates typically range from 1.0 to 1.5%. Prevalence rates vary in studies across Asia, including India and Japan, with India reporting rates ranging from 1.2 to 2.0% and Japan reporting rates of approximately 1.8%.

Previous findings have shown that women with diabetes have a higher risk of breast cancer than women without diabetes [16]. This finding is particularly relevant to the Diabetic Cancerous group of the present study, where 96% of the participants are female. The current study’s findings are consistent with several previous studies that have explored the association between body weight, diabetes mellitus, and breast cancer. For instance, one study found that individuals with both diabetes and breast cancer often exhibited higher body weights, with notable clusters around 70 kg and 80 kg, similar to our findings. This alignment indicates a general trend where increased body weight may contribute to heightened risks of both diseases through mechanisms such as insulin resistance and hormonal dysregulation [17]. Conversely, another study focusing on breast cancer patients without diabetes noted a predominance of lower body weights, paralleling the pattern observed in our Cancerous group alone [18].

A meta-analysis showed a 2% increase in breast cancer risk for every 5 kg/m² increase in BMI [19], which is consistent with our statistically significant findings (*P* < 0.014). In this study, fat accumulation was assessed using standard anthropometric methods, including BMI, waist-to-hip ratio (WHR), and circumferential measurements of the waist, hip, arm, and shoulder, following standardized methods used in South Asian cancer risk studies [20], and current findings indicated widespread abdominal obesity. Central adiposity, in particular, has been associated with both increased breast cancer risk and mortality, especially in premenopausal women [21].

Metabolic syndrome (MetS) in current study was identified using the NCEP ATP III criteria, which require the presence of at least three of the following: elevated waist circumference (≥ 88 cm for women, ≥ 102 cm for men), high fasting glucose (≥ 100 mg/dL), elevated triglycerides (≥ 150 mg/dL), low HDL cholesterol (< 50 mg/dL for women), and hypertension (≥ 130/85 mmHg). A case-control study at Assiut University reported a higher MetS prevalence among breast cancer cases (57.14%) compared to controls (28.6%), with a strong correlation (OR: 33.33, 95% CI 1.91–5.81) [22], supporting our finding of a significant association (*P* < 0.0001) between MetS and disease groups.

Consumption of fried fish (*P* < 0.001), red meat (*P* < 0.0001), and processed meat (*P* < 0.0001). These results are aligned with previous studies that attribute increased breast cancer risks to carcinogens like heterocyclic aromatic amines (HAAs) and polycyclic aromatic hydrocarbons (PAHs) formed during high-temperature cooking and increase breast cancer risk by 5–7% [23]. In current study, egg intake is highest in the Diabetic Cancerous group (58%) with a significant association (*P* < 0.007). This supports evidence that frequent egg consumption, especially when cooked at high temperatures, may raise cancer risk due to cholesterol oxidation and heterocyclic amines [24]. Similarly, the link between high-fat fast foods and disease aligns with findings on sodium nitrates in processed items acting as carcinogens [25].

Furthermore, the frequent intake of instant noodles and pickled items among the Diabetic group is significantly associated with disease status (χ² = 29.84 and 29.77, *P* < 0.0001). These findings correspond to prior research identifying nitrates and sodium in processed foods as risk enhancers [26]. On the contrary, our data revealed that dietary habits such as fruit, root vegetable, and nut consumption are negatively associated with disease status (*P* > 0.306, *P* > 0.349, and *P* > 0.201, respectively), aligning with previous literature that links these foods to protective health outcomes [27]. The inverse associations with tea and coffee (*P* > 0.646 and *P* > 0.695) are also in agreement with prior research indicating that moderate consumption may reduce estrogen receptor-negative breast cancer risk, especially in postmenopausal women [28].

Current research shows no significant associations between food color consumption, aluminium utensil usage, and microwave usage with health status. Similar to current findings, a cross-sectional survey with 500 participants and a study on aluminium utensil usage (P-value 0.135) also found no significant association with health outcomes. These results suggest that the variation observed in these areas is likely random and does not reflect meaningful associations. However, a case-control study examining aluminium foil usage reported a borderline significant association (P-value 0.051), which mirror our findings and suggest a potential trend worth further exploration. This highlights the importance of continuing research to better understand the role of such variables.

Moreover, a cross-sectional study with 500 participants from diverse backgrounds reported no significant association between microwave usage frequency and health status, with a P-value of 0.480, this previous study and current study both showed that microwave usage frequency does not differ significantly across health status groups, which requires further investigation. In contrast, the current study found a significant association between overcooked oil usage and health status (*P* < 0.003), aligning with the results of a cohort study involving 600 participants (P-value 0.002). This suggests that overcooked oil may have a more pronounced effect on health and warrants further investigation.

Research highlights a protective effect of physical activity on breast cancer risk, with a 25% risk reduction for active individuals and a 28% for those engaging in ≥ 6.5 MET-h/week [29], current findings support existing evidence and showing a significant association (*P* < 0.0001) between disease groups and sedentary lifestyle. Sleep deficiency was significantly associated with breast cancer risk in a large study (p-trend ≤ 0.002) [30], although no direct association was found in our study, suggesting future research potential. The effect of digital screen exposure, particularly blue light, has been a topic of emerging concern. Current research shows a significant association (*P* < 0.0001) between prolonged exposure to mobile phone screens and breast cancer risk aligned with previous (*p* = 0.038) [31]. This is similar to other findings, which point to the importance of minimizing screen exposure to reduce potential risks.

While lifestyle factors like smoking and alcohol consumption are not significant in this research, meta-analyses show smoking increases estrogen receptor-positive breast cancer risk (OR = 1.13, *p* < 0.001) [32], this difference in results is seen to be due to cultural differences but future research is needed. The current study supports the potential risk of underarm cosmetic products (*P* < 0.003), which is related to a previous study that found a significant association between aluminum exposure especially at younger ages and increased breast cancer risk (OR = 3.88) [33]. These findings illustrate the complexity of the relationship between lifestyle behaviors and cancer risk, suggesting that the effect may be multifaceted and subject to various moderating factors.

Occupational exposure to chemicals, dust, radiation, and industrial processes is a known risk factor for cancer, in which chemical oncogenes cause mutations that lead to uncontrolled cell growth [34]. The current study is consistent with previous research, which showed a risk of breast cancer incidence among women working in the chemical and non-metallic mineral industries, while a lower incidence was noted in agriculture [35]. Passive smoking also increases the risk of breast cancer, particularly in premenopausal women (OR = 1.29, *p* < 0.001), but not in postmenopausal women (OR = 1.13, *p* = 0.218) [36]. However, this study did not show a significant association, possibly due to underreporting, lower exposure levels, or cultural differences in smoking habits within the Pakistani population.

Current research finds no significant association (*P* > 0.505) between polycystic ovary syndrome and disease risk, aligned with odds ratios from case-control studies (0.87; 95% CI, 0.44 to 1.31) and cohort studies (1.18; 95% CI, 0.93 to 1.43) [37]. Furthermore, while oral contraceptive use is associated (*P* < 0.032) with a modest increase in breast cancer risk, it is consistent with findings from previous studies, such as the Nurses’ Health Study, which found that a slight increase in breast cancer risk among women who used oral contraceptives, specifically triphasic pills [38]. Furthermore, while a history of gestational diabetes mellitus (GDM) alone is not associated with breast cancer in the Sister Study (HR = 1.10, 95% CI = 0.88–1.36), having two or more GDM pregnancies increased breast cancer risk in future, particularly for estrogen receptor-positive breast cancer (HR = 1.81, 95% CI = 1.10–2.98) [39], align with current study result (*P* < 0.0001).

Finally, the relationship between skincare product use and breast cancer risk was explored in a study of black and white women. Among white women, moderate and frequent use of beauty and skincare products was associated with a slightly elevated risk of breast cancer (HR = 1.13 and HR = 1.15, respectively) [(3)], current study shows a significant association (*P* < 0.0001). These findings highlight the complex nature of diabetes and breast cancer association, with varying associations based on product use, contraceptive types, and prior health conditions.

## Strengths and limitations of this study

This study offers several strengths, particularly its focus on the underexplored association between diabetes and breast cancer within a South Asian cohort, which provides important regional insights. A comprehensive assessment of multiple risk factors advances the understanding of these conditions, while strict adherence to ethical standards and the use of structured data collection tool increase the reliability of the results. However, the cross-sectional design limits our ability to establish causal relationships between risk factors and disease outcomes, highlighting the need for longitudinal studies to track changes over time and confirm causality. Additionally, the relatively small sample size may reduce statistical power and limit the generalizability of the results. Furthermore, the limited geographic and demographic diversity of the sample limits broader applicability. Future research should include diverse demographic variables such as socioeconomic status, ethnicity, and rural versus urban residence to better capture variations in risk profiles.

A significant gap in this study is the lack of detailed assessment of dietary habits and traditional cooking methods, which can substantially influence nutrient intake and exposure to harmful compounds. A deep understanding of these cultural practices is critical for accurately assessing their impact on disease risk. Finally, multidisciplinary collaboration involving nutritionists, epidemiologists, geneticists, and clinicians can improve study design, data analysis, and care strategies. Addressing these limitations will strengthen the evidence base, guide targeted prevention efforts, and ultimately help reduce the burden of these diseases.

## Conclusion

This study demonstrates a significant association between diabetes mellitus and breast cancer in the Pakistani population, with notable links to obesity, high intake of red and processed meats, refined sugars, deep-frying cooking methods, sedentary behavior, environmental exposures, and female-specific hormonal and reproductive factors. These findings highlight the complex multifactorial interplay of metabolic, nutritional, lifestyle, and gender-related determinants in the co-development of both diseases. The results underscore the need for culturally tailored public health strategies emphasizing early detection, dietary and lifestyle modifications, environmental risk reduction, and integrated management of diabetes and breast cancer. These insights provide critical evidence to support targeted interventions and inform health policymaking at both community and national levels.

## Supplementary Information

Below is the link to the electronic supplementary material.

Supplementary Material 1

Supplementary Material 2

Supplementary Material 3

## Data Availability

The raw data is available with the corresponding authors, which can be accessed, by requesting them through email.
