# Supplementary material for: Association of diabetes mellitus and breast cancer in adult men and women: a cross-sectional survey
Source: BMC Cancer. 2025 Aug 7;25:1276. doi: 10.1186/s12885-025-14689-6 (PMC12329997; doi:10.1186/s12885-025-14689-6)
Supplement: Supplementary file 1 — Supplementary Material 1 [file 12885_2025_14689_MOESM1_ESM.pdf]

CONSENT FORM FOR PARTICIPATION IN RESEARCH SURVEY

Title of the Study: Association of Diabetes Mellitus and Breast Cancer in Adults: A Cross-Sectional Survey

Principal Investigator: Rabiya Saroosh

Institution: The University of Faisalabad

Contact Information: rabiyasaroosh@gmail.com

Introduction

You are invited to participate in a research study conducted by Rabiya Saroosh from The University of Faisalabad. The purpose of this study is to investigate the association between diabetes mellitus and breast cancer in adults. Your participation is voluntary and will contribute valuable insights to the study.

Purpose of the Study

The objective of this study is to examine the relationship between diabetes mellitus and breast cancer in adult individuals. This research aims to identify any correlations and risk factors that may exist between these two conditions.

Procedures

If you agree to participate in this study, you will be asked to complete a survey. The survey will include questions about your medical history, lifestyle, dietary habits, and other relevant information. The survey will take approximately 20-25 min to complete.

Risks and Benefits

Risks: The risks associated with participating in this study are minimal. You may feel uncomfortable answering some personal or medical questions. You are free to tell.

Benefits: While there may be no direct benefit to you, your participation will help advance our understanding of the relationship between diabetes mellitus and breast cancer. This could potentially lead to better prevention and treatment strategies in the future.

Confidentiality

Your responses will be kept confidential. Data will be stored securely and only accessible to the research team. Your identity will not be disclosed in any reports or publications resulting from this study.

Contact Information

If you have questions at any time about this study, or you experience adverse effects as the result of participating in this study, you may contact the researcher whose contact information is provided on the first page

Voluntary Participation

Your participation in this study is voluntary. It is up to you to decide whether or not to take part in this study. If you decide to take part in this study, you will be asked to sign a consent form. After you sign the consent form, you are still free to withdraw at any time and without giving a reason. Withdrawing from this study will not affect the relationship you have, if any, with the researcher. If you withdraw from the study before data collection is completed, your data will be returned to you or destroyed.

Consent

I have read and I understand the provided information and have had the opportunity to ask questions. I understand that my participation is voluntary and that I am free to withdraw at any time, without giving a reason and without cost. I understand that I will be given a copy of this consent form. I voluntarily agree to take part in this study.

Participant's signature ______________________________Date __________

Researcher's signature ______________________________ Date __________
