# Supplementary material for: Association of diabetes mellitus and breast cancer in adult men and women: a cross-sectional survey
Source: BMC Cancer. 2025 Aug 7;25:1276. doi: 10.1186/s12885-025-14689-6 (PMC12329997; doi:10.1186/s12885-025-14689-6)
Supplement: Supplementary file 2 — Supplementary Material 2 [file 12885_2025_14689_MOESM2_ESM.docx]

| **Table 1.** Criteria for the diagnosis of diabetes mellitus (15) |
| --- |
| - A1C ≥ 6.5 % (48 m mol/mol)   OR   - 2-h plasma glucose ≥ 200 mg/dL (11.1 mmol/L)   OR   - Fasting plasma glucose ≥126 mg/dL (7.0 mmol/L)   OR   - Random plasma glucose ≥ 200 mg/dL (11.1 mmol/L) |

| **Table 2.** Criteria for the diagnosis of breast cancer (16) |
| --- |
| - Diagnosis confirmed through mammography, ultrasound, or biopsy   OR   - Clinical staging based on the TNM (Tumor, Node, Metastasis) classification system   OR   - Histologically confirmed diagnosis of invasive breast carcinoma, including ductal carcinoma in situ (DCIS) and invasive ductal carcinoma (IDC) |

**Table 3.** Relationship between study groups and gender distribution

| **Parameter** | **Groups** | | | | | **Chi-square**  **(P-value)** |
| --- | --- | --- | --- | --- | --- | --- |
|  | **Normal (%) n=100** | **Diabetic (%) n=100** | **Cancerous (%) n=100** | **Diabetic Cancerous (%) n=100** | **Total (%) n=400** |  |
| **Gender** | | | | | | |
| Male | 49 (49%) | 13 (13%) | 0 (0%) | 4 (4%) | 66 (16.5%) | 108.65^**^  P<0.0001 |
| Female | 51 (51%) | 87 (87%) | 100 (100%) | 96 (96%) | 334 (83.5%) |  |

**NS = Non-significant (P>0.05); * = Significant (P<0.05); ** = Highly significant (P<0.01)**

**Table 4.** Comparison of weight distribution across study groups using ANOVA

| **Parameters** | **Sum of Squares** | **df** | **Mean Square** | **F** | **Sig.** |
| --- | --- | --- | --- | --- | --- |
| Weight | 2996.010 | 3 | 998.670 | 3.918 | 0.009**^**^** |

**NS = Non-significant (P>0.05); * = Significant (P<0.05); ** = Highly significant (P<0.01)**

**Table 5.** Association of clinical and medical history with different health status groups among respondents

| **Parameters** | **Groups** | | | | | **Chi-square**  **(P-value)** | |
| --- | --- | --- | --- | --- | --- | --- | --- |
|  | **Normal (%) n=100** | **Diabetic (%) n=100** | **Cancerous (%) n=100** | **Diabetic Cancerous (%) n=100** | **Total (%) n=400** |  |  |
| **BMI** | | | | | | | |
| Underweight | 4 (4%) | 0 (0%) | 5 (5%) | 1 (1%) | 10 (2.5%) | | 20.68^*^  P**<**0.014 |
| Normal weight | 36 (36%) | 30 (30%) | 37 (37%) | 31 (31%) | 134 (33.5%) | |  |
| Overweight | 37 (37%) | 29 (29%) | 18 (18%) | 28 (28%) | 112 (28%) | |  |
| Obese | 23 (23%) | 41 (41%) | 40 (40%) | 40 (40%) | 144 (36%) | |  |
| **Fat Accumulation Areas** | | | | | | | |
| Abdomen | 44 (44%) | 32 (32%) | 30 (30%) | 31 (31%) | 137 (34.2%) | 103.21**  P<0.0001 | |
| Buttocks | 1 (1%) | 2 (2%) | 2 (2%) | 6 (6%) | 11 (2.8%) |  |  |
| Thighs | 7 (7%) | 4 (4%) | 0 (0%) | 4 (4%) | 15 (3.8%) |  |  |
| Shoulders | 0 (0%) | 0 (0%) | 0 (0%) | 1 (1%) | 1 (0.2%) |  |  |
| Abdomen, Breast | 0 (0%) | 3 (3%) | 0 (0%) | 0 (0%) | 3 (0.8%) |  |  |
| Abdomen, Buttocks | 1 (1%) | 6 (6%) | 2 (2%) | 1 (1%) | 10 (2.5%) |  |  |
| Abdomin, Shoulders | 0 (0%) | 3 (3%) | 0 (0%) | 0 (0%) | 3 (0.8%) |  |  |
| Abdomin, Thighs | 9 (9%) | 16 (16%) | 13 (13%) | 20 (20%) | 58 (14.5%) |  |  |
| Thighs, Buttocks | 5 (5%) | 2 (2%) | 2 (2%) | 3 (3%) | 12 (3%) |  |  |
| Abdomen, Buttocks, Shoulders | 1 (1%) | 0 (0%) | 0 (0%) | 0 (0%) | 1 (0.2%) |  |  |
| Abdomen, Thigh, Breast | 0 (0%) | 3 (3%) | 0 (0%) | 0 (0%) | 3 (0.8%) |  |  |
| Abdomen, Thighs, Buttocks | 4 (4%) | 4 (4%) | 18 (18%) | 8 (8%) | 34 (8.5%) |  |  |
| Abdomin, Thighs, Shoulders | 0 (0%) | 2 (2%) | 0 (0%) | 0 (0%) | 2 (0.5%) |  |  |
| Abdomen, Breast, Thighs, Buttocks | 0 (0%) | 1 (1%) | 0 (0%) | 0 (0%) | 1 (0.2%) |  |  |
| Abdomen, Thighs, Buttocks, Breast | 0 (0%) | 2 (2%) | 1 (1%) | 0 (0%) | 3 (0.8%) |  |  |
| Abdomen, Thighs, Buttocks, Shoulders | 0 (0%) | 0 (%) | 0 (0%) | 3 (3%) | 3 (0.8%) |  |  |
| No | 28 (28%) | 20 (20%) | 32 (32%) | 23 (23%) | 103 (25.8%) |  |  |
| **Metabolic syndrome** | | | | | | | |
| No | 61 (61%) | 28 (28%) | 51 (51%) | 36 (36%) | 176 (44%) | 26.71^**^  P**<0.0001** | |
| Yes | 39 (39%) | 72 (72%) | 49 (49%) | 64 (64%) | 224 (56%) |  |  |

**NS = Non-significant (P>0.05); * = Significant (P<0.05); ** = Highly significant (P<0.01)**

**Table 6.** Association of dietary habits with different health status groups among respondents

| **Parameters** | **Groups** | | | | | | | **Chi-square**  **(P-value)** | |
| --- | --- | --- | --- | --- | --- | --- | --- | --- | --- |
|  | **Normal (%) n=100** | | | **Diabetic (%) n=100** | **Cancerous (%) n=100** | **Diabetic Cancerous (%) n=100** | **Total (%) n=400** |  |  |
| **Fried Fish** | | | | | | | | | |
| Weekly | 10 (10%) | | | 11 (11%) | 5 (5%) | 5 (5%) | 31 (7.8%) | 32.99^**^  P<0.0001 | |
| Occasionally | 83 (83%) | | | 66 (66%) | 67 (67%) | 81 (81%) | 297 (74.2%) |  |  |
| Used to | 5 (5%) | | | 14 (14%) | 7 (7%) | 4 (4%) | 30 (7.5%) |  |  |
| Never | 2 (2%) | | | 9 (9%) | 21 (21%) | 10 (10%) | 42 (10.5%) |  |  |
| **Red meat** | | | | | | | | | |
| Weekly | 25 (25%) | | | 18 (18%) | 16 (16%) | 14 (14%) | 73 (18.2%) | 32.71^**^  P<0.0001 | |
| Occasionally | 66 (66%) | | | 60 (60%) | 55 (55%) | 72 (72%) | 253 (63.2%) |  |  |
| Used to | 4 (4%) | | | 16 (16%) | 8 (8%) | 6 (6%) | 34 (8.5%) |  |  |
| Never | 5 (5%) | | | 6 (6%) | 21 (21%) | 8 (8%) | 40 (10%) |  |  |
| **Processed meat** | | | | | | | | | |
| Weekly | | | 18 (18%) | 12 (12%) | 4 (4%) | 3 (3%) | 37 (9.2%) | 43.20^**^  P<0.0001 | |
| Occasionally | | | 63 (63%) | 53 (53%) | 42 (42%) | 63 (63%) | 221 (55.2%) |  |  |
| Used to | | | 2 (2%) | 9 (9%) | 8 (8%) | 3 (3%) | 22 (5.5%) |  |  |
| Never | | | 17 (17%) | 26 (26%) | 46 (46%) | 31 (31%) | 120 (30%) |  |  |
| **Meat or poultry** | | | | | | | | | |
| Weekly | | | 63 (63%) | 45 (45%) | 61 (61%) | 56 (56%) | 225 (56.2%) | 30.75^**^  P<0.0001 | |
| Occasionally | | | 34 (34%) | 40 (40%) | 27 (27%) | 42 (42%) | 143 (35.8%) |  |  |
| Used to | | | 1 (1%) | 12 (12%) | 5 (5%) | 2 (2%) | 20 (5%) |  |  |
| Never | | | 2 (2%) | 3 (3%) | 7 (7%) | 0 (0%) | 12 (3%) |  |  |
| **Egg** | | | | | | | | | |
| Weekly | | | 57 (57%) | 40 (40%) | 54 (54%) | 58 (58%) | 209 (52.2%) | 22.54^**^  P<0.007 | |
| Occasionally | | | 33 (33%) | 38 (38%) | 38 (38%) | 35 (35%) | 144 (36%) |  |  |
| Used to | | | 10 (10%) | 17 (17%) | 8 (8%) | 6 (6%) | 41 (10.2%) |  |  |
| Never | | | 0 (0%) | 5 (5%) | 0 (0%) | 1 (1%) | 6 (1.5%) |  |  |
| **Fast food (pizza, burger, fries etc.)** | | | | | | | | | |
| Weekly | | | 15 (15%) | 8 (8%) | 5 (5%) | 6 (6%) | 34 (8.5%) | 25.80^**^  P<0.002 | |
| Occasionally | | | 54 (54%) | 45 (45%) | 38 (38%) | 52 (52%) | 189 (47.2%) |  |  |
| Used to | | | 3 (3%) | 13 (13%) | 14 (14%) | 4 (4%) | 34 (8.5%) |  |  |
| Never | | | 28 (28%) | 34 (34%) | 43 (43%) | 38 (38%) | 143 (35.8%) |  |  |
| **Instant noodles** | | | | | | | | | |
| Weekly | | | 1 (1%) | 7 (7%) | 2 (2%) | 1 (1%) | 11 (2.8%) | 29.84^**^  P<0.0001 | |
| Occasionally | | | 24 (24%) | 34 (34%) | 23 (23%) | 25 (25%) | 106 (26.5%) |  |  |
| Used to | | | 3 (3%) | 12 (12%) | 5 (5%) | 2 (2%) | 22 (5.5%) |  |  |
| Never | | | 72 (72%) | 47 (47%) | 70 (70%) | 72 (72%) | 261 (65.2%) |  |  |
| **Pickle** | | | | | | | | | |
| Weekly | | | 41 (41%) | 44 (44%) | 40 (40%) | 28 (28%) | 153 (38.2%) | 29.77^**^  P<0.0001 | |
| Occasionally | | | 45 (45%) | 38 (38%) | 29 (29%) | 47 (47%) | 159 (39.8%) |  |  |
| Used to | | | 10 (10%) | 17 (17%) | 19 (19%) | 23 (23%) | 69 (17.2%) |  |  |
| Never | | | 4 (4%) | 1 (1%) | 12 (12%) | 2 (2%) | 19 (4.8%) |  |  |
| **Bakery products (Rusk, cake rusk, etc)** | | | | | | | | | |
| Weekly | | | 55 (55%) | 38 (38%) | 57 (57%) | 52 (52%) | 202 (50.5%) | 16.42^NS^  P**>**0.059 | |
| Occasionally | | | 45 (45%) | 55 (55%) | 36 (36%) | 44 (44%) | 180 (45%) |  |  |
| Used to | | | 0 (0%) | 4 (4%) | 5 (5%) | 3 (3%) | 12 (3%) |  |  |
| Never | | | 0 (0%) | 3 (3%) | 2 (2%) | 1 (1%) | 6 (1.5%) |  |  |
| **White bread** | | | | | | | | | |
| Weekly | | | 21 (21%) | 24 (24%) | 32 (32%) | 29 (29%) | 106 (26.5%) | 12.94^NS^  P**>**0.165 | |
| Occasionally | | | 56 (56%) | 53 (53%) | 45 (45%) | 53 (53%) | 207 (51.8%) |  |  |
| Used to | | | 3 (3%) | 6 (6%) | 10 (10%) | 2 (2%) | 21 (5.2%) |  |  |
| Never | | | 20 (20%) | 17 (17%) | 13 (13%) | 16 (16%) | 66 (16.5%) |  |  |
| **Homemade butter** | | | | | | | | | |
| Weekly | | | 33 (33%) | 25 (25%) | 18 (18%) | 18 (18%) | 94 (23.5%) | 15.48^NS^  P**>**0.079 | |
| Occasionally | | | 27 (27%) | 27 (27%) | 24 (24%) | 36 (36%) | 114 (28.5%) |  |  |
| Used to | | | 21 (21%) | 27 (27%) | 25 (25%) | 20 (20%) | 93 (23.2%) |  |  |
| Never | | | 19 (19%) | 21 (21%) | 33 (33%) | 26 (26%) | 99 (24.8%) |  |  |
| **Blue band butter** | | | | | | | | | |
| Weekly | | | 1 (1%) | 0 (0%) | 0 (0%) | 1 (1%) | 2 (0.5%) | 11.58^NS^  P**>**0.238 | |
| Occasionally | | | 6 (6%) | 10 (10%) | 5 (5%) | 1 (1%) | 22 (5.5%) |  |  |
| Used to | | | 7 (7%) | 4 (4%) | 7 (7%) | 4 (4%) | 22 (5.5%) |  |  |
| Never | | | 86 (86%) | 86 (86%) | 88 (88%) | 94 (94%) | 354 (88.5%) |  |  |
| **Whole milk** | | | | | | | | | |
| Weekly | | | 30 (30%) | 28 (28%) | 28 (28%) | 21 (21%) | 107 (26.8%) | 13.99^NS^  P**>**0.123 | |
| Occasionally | | | 6 (6%) | 5 (5%) | 3 (3%) | 3 (3%) | 17 (4.2%) |  |  |
| Used to | | | 2 (2%) | 9 (9%) | 3 (3%) | 2 (2%) | 16 (4%) |  |  |
| Never | | | 62 (62%) | 58 (58%) | 66 (66%) | 74 (74%) | 260 (65%) |  |  |
| **Coffee** | | | | | | | | | |
| Weekly | | | 10 (10%) | 5 (5%) | 4 (4%) | 5 (5%) | 24 (6%) | 6.44^NS^  P>0.695 | |
| Occasionally | | | 15 (15%) | 15 (15%) | 14 (14%) | 16 (16%) | 60 (15%) |  |  |
| Used to | | | 5 (5%) | 10 (10%) | 6 (6%) | 9 (9%) | 30 (7.5%) |  |  |
| Never | | | 70 (70%) | 70 (70%) | 76 (76%) | 70 (70%) | 286 (71.5%) |  |  |
| **Tea** | | | | | | | | | |
| Weekly | | | 88 (88%) | 92 (92%) | 92 (92%) | 91 (91%) | 363 (90.8%) | 6.92^NS^  P**>**0.646 | |
| Occasionally | | | 9 (9%) | 8 (8%) | 6 (6%) | 6 (6%) | 29 (7.2%) |  |  |
| Used to | | | 2 (2%) | 0 (0%) | 2 (2%) | 1 (1%) | 5 (1.2%) |  |  |
| Never | | | 1 (1%) | 0 (0%) | 0 (0%) | 2 (2%) | 3 (0.8%) |  |  |
| **Tea whitener** | | | | | | | | | |
| Weekly | | | 3 (3%) | 3 (3%) | 2 (2%) | 2 (2%) | 10 (2.5%) | 9.40^NS^  P**>**0.401 | |
| Occasionally | | | 16 (16%) | 21 (21%) | 8 (8%) | 16 (16%) | 61 (15.2%) |  |  |
| Used to | | | 24 (24%) | 20 (20%) | 26 (26%) | 17 (17%) | 87 (21.8%) |  |  |
| Never | | | 57 (57%) | 56 (56%) | 64 (64%) | 65 (65%) | 242 (60.5%) |  |  |
| **Soft drinks** | | | | | | | | | |
| Weekly | | | 30 (30%) | 13 (13%) | 19 (19%) | 24 (24%) | 86 (21.5%) | 19.15^*^  P**<**0.024 | |
| Occasionally | | | 60 (60%) | 57 (57%) | 62 (62%) | 56 (56%) | 235 (58.8%) |  |  |
| Used to | | | 8 (8%) | 22 (22%) | 16 (16%) | 14 (14%) | 60 (15%) |  |  |
| Never | | | 2 (2%) | 8 (8%) | 3 (3%) | 6 (6%) | 19 (4.8%) |  |  |
| **Processed fruit-juice** | | | | | | | | | |
| Weekly | | | 9 (9%) | 9 (9%) | 8 (8%) | 5 (5%) | 31 (7.8%) | 27.98^**^  P**<**0.001 | |
| Occasionally | | | 54 (54%) | 49 (49%) | 65 (65%) | 49 (49%) | 217 (54.2%) |  |  |
| Used to | | | 8 (8%) | 21 (21%) | 17 (17%) | 10 (10%) | 56 (14%) |  |  |
| Never | | | 29 (29%) | 21 (21%) | 10 (10%) | 36 (36%) | 96 (24%) |  |  |
| **Citrus fruit juices** | | | | | | | | | |
| Weekly | | | 19 (19%) | 9 (9%) | 23 (23%) | 11 (11%) | 62 (15.5%) | 25.70^**^  P**<**0.002 | |
| Occasionally | | | 50 (50%) | 36 (36%) | 48 (48%) | 43 (43%) | 177 (44.2%) |  |  |
| Used to | | | 2 (2%) | 11 (11%) | 5 (5%) | 6 (6%) | 24 (6%) |  |  |
| Never | | | 29 (29%) | 44 (44%) | 24 (24%) | 40 (40%) | 137 (34.2%) |  |  |
| **Fruits** | | | | | | | | | |
| Weekly | | | 76 (76%) | 78 (78%) | 85 (85%) | 75 (75%) | 314 (78.5%) | 3.6^NS^  P>0.306 | |
| Occasionally | | | 24 (24%) | 22 (22%) | 15 (15%) | 25 (25%) | 86 (21.5%) |  |  |
| Used to | | | 0 (0%) | 0 (0%) | 0 (0%) | 0 (0%) | 0 (0%) |  |  |
| Never | | | 0 (0%) | 0 (0%) | 0 (0%) | 0 (0%) | 0 (0%) |  |  |
| **Root vegetables (shljm, chukndr, adrk, alo)** | | | | | | | | | |
| Weekly | | | 100 (100%) | 98 (98%) | 100 (100%) | 98 (98%) | 396 (99%) | 6.7^NS^  P>0.349 | |
| Occasionally | | | 0 (0%) | 2 (2%) | 0 (0%) | 1 (1%) | 3 (0.8%) |  |  |
| Used to | | | 0 (0%) | 0 (0%) | 0 (0%) | 1 (1%) | 1 (0.2%) |  |  |
| Never | | | 0 (0%) | 0 (0%) | 0 (0%) | 0 (0%) | 0 (0%) |  |  |
| **Leafy vegetables (gobi, plk, salad ky pty)** | | | | | | | | | |
| Weekly | | | 75 (75%) | 70 (70%) | 80 (80%) | 75 (75%) | 300 (75%) | 2.667^NS^  P>0.446 | |
| Occasionally | | | 25 (25%) | 30 (30%) | 20 (15%) | 25 (25%) | 100 (25%) |  |  |
| Used to | | | 0 (0%) | 0 (0%) | 0 (0%) | 0 (0%) | 0 (0%) |  |  |
| Never | | | 0 (0%) | 0 (0%) | 0 (0%) | 0 (0%) | 0 (0%) |  |  |
| **Lentils** | | | | | | | | | |
| Weekly | | | 70 (70%) | 65 (65%) | 68 (68%) | 65 (65%) | 268 (67%) | 0.814^NS^  P>0.846 | |
| Occasionally | | | 30 (30%) | 35 (35%) | 32 (32%) | 35 (35%) | 132 (33%) |  |  |
| Used to | | | 0 (0%) | 0 (0%) | 0 (0%) | 0 (0%) | 0 (0%) |  |  |
| Never | | | 0 (0%) | 0 (0%) | 0 (0%) | 0 (0%) | 0 (0%) |  |  |
| **White rice** | | | | | | | | | |
| Weekly | | | 67 (67%) | 62 (62%) | 61 (61%) | 66 (66%) | 256 (64%) | 3.9^NS^  P**>**0.689 | |
| Occasionally | | | 29 (29%) | 28 (28%) | 33 (33%) | 28 (28%) | 118 (29.5%) |  |  |
| Used to | | | 4 (4%) | 10 (10%) | 6 (6%) | 6 (6%) | 26 (6.5%) |  |  |
| Never | | | 0 (0%) | 0 (0%) | 0 (0%) | 0 (0%) | 0 (0%) |  |  |
| **White flour** | | | | | | | | | |
| Weekly | | | 15 (15%) | 7 (7%) | 6 (6%) | 5 (5%) | 33 (8.2%) | 43.68^**^  P**<0.0001** | |
| Occasionally | | | 70 (70%) | 83 (83%) | 52 (52%) | 75 (75%) | 280 (70%) |  |  |
| Used to | | | 3 (3%) | 2 (2%) | 10 (10%) | 7 (7%) | 22 (5.5%) |  |  |
| Never | | | 12 (12%) | 8 (8%) | 32 (32%) | 13 (13%) | 65 (16.2%) |  |  |
| **Nuts** | | | | | | | | | |
| Weekly | | | 27 (27%) | 19 (19%) | 17 (17%) | 27 (27%) | 90 (22.5%) | 12.22^NS^  P**>**0.201 | |
| Occasionally | | | 63 (63%) | 63 (63%) | 75 (75%) | 59 (59%) | 260 (65%) |  |  |
| Used to | | | 4 (4%) | 10 (10%) | 6 (6%) | 8 (8%) | 28 (7%) |  |  |
| Never | | | 6 (6%) | 8 (8%) | 2 (2%) | 6 (6%) | 22 (5.5%) |  |  |
| **White sugar** | | | | | | | | | |
| Weekly | | | 82 (82%) | 52 (52%) | 84 (84%) | 38 (38%) | 256 (64%) | 86.47^**^  P<0.0001 | |
| Occasionally | | | 17 (17%) | 34 (34%) | 10 (10%) | 57 (57%) | 118 (29.5%) |  |  |
| Used to | | | 1 (1%) | 14 (14%) | 6 (6%) | 4 (4%) | 25 (6.2%) |  |  |
| Never | | | 0 (0%) | 0 (0%) | 0 (0%) | 1 (1%) | 1 (0.2%) |  |  |
| **Ice cream** | | | | | | | | | |
| Weekly | | | 10 (10%) | 3 (3%) | 11 (11%) | 2 (2%) | 26 (6.5%) | 26.20^**^  P<0.002 | |
| Occasionally | | | 73 (73%) | 78 (78%) | 58 (58%) | 71 (71%) | 280 (70%) |  |  |
| Used to | | | 8 (8%) | 15 (15%) | 12 (12%) | 14 (14%) | 49 (12.2%) |  |  |
| Never | | | 9 (9%) | 4 (4%) | 19 (19%) | 13 (13%) | 45 (11.2%) |  |  |
| **Chocolate** | | | | | | | | | |
| Weekly | | | 12 (12%) | 7 (7%) | 8 (8%) | 3 (3%) | 30 (7.5%) | 14.05^NS^  P>0.121 | |
| Occasionally | | | 39 (39%) | 39 (39%) | 42 (42%) | 48 (48%) | 168 (42%) |  |  |
| Used to | | | 13 (13%) | 24 (24%) | 18 (18%) | 11 (11%) | 66 (16.5%) |  |  |
| Never | | | 36 (36%) | 30 (30%) | 32 (32%) | 38 (38%) | 136 (34%) |  |  |
| **Deep-fried food items** | | | | | | | | | |
| Weekly | | | 31 (31%) | 4 (4%) | 9 (9%) | 10 (10%) | 54 (13.5%) | | 52.38^**^  P**<0.0001** |
| Occasionally | | | 63 (63%) | 95 (95%) | 79 (79%) | 78 (78%) | 315 (78.8%) | |  |
| Used to | | | 2 (2%) | 1 (1%) | 7 (7%) | 9 (9%) | 19 (4.8%) | |  |
| Never | | | 4 (4%) | 0 (0%) | 5 (5%) | 3 (3%) | 12 (3%) | |  |
| **Salt intake** | | | | | | | | | |
| 1-2 spoons | | 99 (99%) | | 100 (100%) | 98 (98%) | 98 (98%) | 395 (98.8%) | | 2.23^NS^  P**>**0.526 |
| 3-4 spoons | | 1 (1%) | | 0 (0%) | 2 (2%) | 2 (2%) | 5 (1.2%) | |  |
| 5-6 spoons | | 0 (0%) | | 0 (0%) | 0 (0%) | 0 (0%) | 0 (0%) | |  |
| **Avoid fat while eating meat** | | | | | | | | | |
| Yes | | 72 (72%) | | 80 (80%) | 75 (75%) | 80 (80%) | 307 (76.8%) | | 4.556^NS^  P**>**0.602 |
| No | | 21 (21%) | | 18 (18%) | 21 (21%) | 16 (16%) | 76 (19%) | |  |
| Sometimes | | 7 (7%) | | 2 (2%) | 4 (4%) | 4 (4%) | 17 (4.2%) | |  |
| **Follow any specific diet** | | | | | | | | | |
| No | | 100 (100%) | | 100 (100%) | 100 (100%) | 100 (100%) | 400 (100%) | | **-** |
| Mediterranean diet | | 0 (0%) | | 0 (0%) | 0 (0%) | 0 (0%) | 0 (0%) | |  |
| Vegetarian diet | | 0 (0%) | | 0 (0%) | 0 (0%) | 0 (0%) | 0 (0%) | |  |
| Vegan diet | | 0 (0%) | | 0 (0%) | 0 (0%) | 0 (0%) | 0 (0%) | |  |
| Ketogenic diet | | 0 (0%) | | 0 (0%) | 0 (0%) | 0 (0%) | 0 (0%) | |  |
| Non-vegetarian | | 0 (0%) | | 0 (0%) | 0 (0%) | 0 (0%) | 0 (0%) | |  |
| Other | | 0 (0%) | | 0 (0%) | 0 (0%) | 0 (0%) | 0 (0%) | |  |

**NS = Non-significant (P>0.05); * = Significant (P<0.05); ** = Highly significant (P<0.01)**

**Table 7.** Descriptive statistics of respondents' eating habits and cooking practices across study groups: frequency of food item consumption

| **Food Items** | **Groups** | | | | | | | | | | | | | |
| --- | --- | --- | --- | --- | --- | --- | --- | --- | --- | --- | --- | --- | --- | --- |
|  | **Normal** | | | **Diabetic** | | | | | **Cancerous** | | | **Diabetic Cancerous** | | |
|  | **N** | **Mean** | **SE** | | **N** | **Mean** | | **SE** | **N** | **Mean** | **SE** | **N** | **Mean** | **SE** |
| Fish | 10 | 1.70 | .40 | | 11 | | 2.09 | .39 | 5 | 1.80 | .37 | 5 | 1.60 | .24 |
| Red meat | 25 | 1.52 | .15 | | 18 | | 1.39 | .20 | 16 | 1.62 | .20 | 14 | 1.79 | .42 |
| Processed meat | 18 | 1.83 | .35 | | 12 | | 1.42 | .19 | 4 | 1.00 | .00 | 3 | 2.00 | .58 |
| Meat or poultry | 63 | 2.13 | .19 | | 45 | | 2.49 | .26 | 61 | 1.92 | .19 | 56 | 1.71 | .14 |
| Eggs | 57 | 3.68 | .31 | | 40 | | 3.17 | .32 | 54 | 3.54 | .31 | 58 | 3.48 | .32 |
| Fast food | 15 | 1.80 | .28 | | 8 | | 1.63 | .26 | 5 | 1.00 | .00 | 6 | 1.17 | .17 |
| Instant noodles | 1 | 1.00 | . | | 7 | | 1.57 | .30 | 2 | 1.00 | .00 | 1 | 1.00 | . |
| Pickles | 41 | 3.02 | .33 | | 44 | | 3.09 | .30 | 40 | 3.03 | .36 | 28 | 3.54 | .48 |
| Bakery products | 55 | 3.33 | .31 | | 38 | | 4.26 | .37 | 57 | 3.32 | .29 | 52 | 3.63 | .33 |
| White bread | 21 | 2.38 | .41 | | 24 | | 3.42 | .54 | 32 | 2.34 | .30 | 29 | 2.00 | .34 |
| Homemade butter | 33 | 5.21 | .43 | | 24 | | 5.50 | .49 | 18 | 4.89 | .60 | 18 | 5.94 | .48 |
| Blueband butter | 1 | 1.00 | . | | 0 | | . | . | 0 | . | . | 1 | 3.00 | . |
| Drink whole milk | 30 | 6.87 | .13 | | 28 | | 6.79 | .21 | 28 | 6.64 | .25 | 21 | 6.76 | .24 |
| Drink coffee | 10 | 5.20 | 2.13 | | 1 | | 1.00 | . | 0 | . | . | 1 | 3.00 | . |
| Drink tea | 88 | 15.00 | .71 | | 92 | | 14.63 | .73 | 92 | 15.52 | .74 | 91 | 13.38 | .63 |
| Tea whitener | 3 | 7.00 | .00 | | 3 | | 2.00 | .00 | 2 | 2.00 | .00 | 2 | 5.00 | 2.00 |
| Soft drinks | 30 | 3.47 | .43 | | 13 | | 2.00 | .45 | 19 | 1.79 | .21 | 24 | 2.83 | .38 |
| Processed fruit juices | 9 | 2.00 | .33 | | 9 | | 2.11 | .26 | 8 | 1.13 | .12 | 5 | 3.00 | 1.00 |
| Citrus fruit juices | 19 | 2.47 | .39 | | 9 | | 1.89 | .31 | 23 | 3.83 | .54 | 11 | 4.00 | .76 |
| Fruits | 76 | 4.34 | .27 | | 78 | | 3.94 | .27 | 85 | 4.86 | .27 | 75 | 5.05 | .28 |
| Root vegetables | 100 | 4.09 | .16 | | 98 | | 4.49 | .16 | 100 | 3.89 | .15 | 98 | 4.04 | .13 |
| Leafy vegetables | 83 | 1.66 | .10 | | 59 | | 1.71 | .16 | 85 | 1.96 | .12 | 75 | 1.76 | .12 |
| Lentils | 89 | 1.76 | .10 | | 77 | | 2.32 | .14 | 94 | 1.59 | .09 | 86 | 1.56 | .08 |
| White rice | 67 | 2.15 | .15 | | 62 | | 1.95 | .15 | 61 | 1.72 | .10 | 66 | 2.02 | .15 |
| White flour | 15 | 2.00 | .54 | | 7 | | 3.14 | .34 | 6 | 1.67 | .33 | 5 | 4.60 | 1.47 |
| Nuts | 27 | 3.89 | .49 | | 19 | | 4.05 | .54 | 17 | 3.41 | .68 | 27 | 5.04 | .48 |
| White sugar | 82 | 7.00 | .00 | | 53 | | 7.00 | .00 | 84 | 7.00 | .00 | 38 | 6.92 | .08 |
| Ice cream | 10 | 1.20 | .13 | | 3 | | 2.00 | .00 | 11 | 3.64 | .82 | 2 | 1.50 | .50 |
| Chocolate | 12 | 3.08 | .61 | | 7 | | 1.86 | .86 | 8 | 4.13 | 1.09 | 3 | 3.33 | 1.86 |
| Deep-fried items | 31 | 2.10 | .29 | | 4 | | 1.00 | .00 | 9 | 1.00 | .00 | 10 | 1.20 | .13 |
| Artificial food color | 12 | 1.83 | .51 | | 3 | | 2.00 | .00 | 9 | 1.00 | .00 | 6 | 1.17 | .17 |
| Aluminum foil | 2 | 1.50 | .50 | | 0 | | . | . | 0 | . | . | 0 | . | . |

**Table 8.** Association of kitchen practices with different health status groups among respondents

| **Kitchen Practices** | **Groups** | | | | | | | **Chi-square**  **(P-value)** |
| --- | --- | --- | --- | --- | --- | --- | --- | --- |
|  | **Normal (%) n=100** | | **Diabetic (%) n=100** | **Cancerous (%) n=100** | **Diabetic Cancerous (%) n=100** | | **Total (%) n=400** |  |
| **Food color** | | | | | | | | |
| Weekly | | 12 (12%) | 3 (3%) | 9 (9%) | 6 (6%) | | 30 (7.5%) | 15.74^NS^  P>0.073 |
| Occasionally | | 70 (70%) | 77 (77%) | 63 (63%) | 70 (70%) | | 280 (70%) |  |
| Used to | | 3 (3%) | 10 (10%) | 8 (8%) | 5 (5%) | | 26 (6.5%) |  |
| Never | | 15 (15%) | 10 (10%) | 20 (20%) | 19 (19%) | | 64 (16%) |  |
| **Aluminum foils are used for cooking/baking/storing** | | | | | | | | |
| Weekly | | 2 (2%) | 0 (0%) | 0 (0%) | 2 (2%) | | 4 (1%) | 16.62**^*^**  P**<**0.055 |
| Occasionally | | 5 (5%) | 6 (6%) | 0 (0%) | 2 (2%) | | 13 (3.2%) |  |
| Used to | | 2 (2%) | 0 (0%) | 3 (3%) | 0 (0%) | | 5 (1.2%) |  |
| Never | | 91 (91%) | 94 (94%) | 97 (97%) | 96 (96%) | | 378 94.5(%) |  |
| **Aluminum utensils are used for cooking** | | | | | | | | |
| Never | | 90 (90%) | 97 (97%) | 94 (94%) | 97 (97%) | | 378 (94.5%) | 9.95^NS^  P**>**0.127 |
| Regularly | | 10 (10%) | 3 (3%) | 6 (6%) | 3 (3%) | | 20 (5%) |  |
| Used to | | 0 (0%) | 0 (0%) | 0 (0%) | 0 (0%) | | 2 (0.5%) |  |
| **Ghee/oil usage for cooking** | | | | | | | | |
| Ghee | | 41(%) | 52(%) | 57(%) | 57(%) | | 207(%) | 10.16^NS^  P**>**0.118 |
| Oil | | 49(%) | 33(%) | 33(%) | 33(%) | | 148(%) |  |
| Both | | 10(%) | 15(%) | 10(%) | 10(%) | | 45(%) |  |
| **Overcooked oil** | | | | | | | | |
| Never | | 19 (19%) | 11 (11%) | 33 (33%) | | 20 (20%) | 83 (20.8%) | 25.17**^**^**  P**<**0.003 |
| Occasionally | | 0 (0%) | 0 (0%) | 3 (3%) | | 1 (1%) | 4 (1%) |  |
| Regularly | | 81 (%) | 89 (89%) | 64 (64%) | | 78 (78%) | 312 (78%) |  |
| Used to | | 0 (0%) | 0 (0%) | 0 (0%) | | 1 (1%) | 1 (0.2%) |  |
| **Repeatedly use the same cooked oil for cooking/frying** | | | | | | | | |
| Never | | 23 (23%) | 19 (19%) | 16 (16%) | | 14 (14%) | 72 (18%) | 9.48^NS^  P**>**0.148 |
| Occasionally | | 77 (77%) | 79 (79%) | 81 (81%) | | 80 (80%) | 317 (79.2%) |  |
| Regularly | | 0 (0%) | 2 (2%) | 3 (3%) | | 6 (6%) | 11 (2.8%) |  |
| **Microwave usage** | | | | | | | | |
| Never | | 49 (49%) | 57 (57%) | 59 (59%) | | 52 (52%) | 217 (54.2%) | 8.51^NS^  P**>**0.484 |
| Used to | | 6 (6%) | 5 (5%) | 6 (6%) | | 5 (5%) | 22 (5.5%) |  |
| Occasionally | | 14 (14%) | 21 (21%) | 16 (16%) | | 16 (16%) | 67 (16.8%) |  |
| Regularly | | 31 (31%) | 17 (17%) | 19 (19%) | | 27 (27%) | 94 (23.5%) |  |

**NS = Non-significant (P>0.05); * = Significant (P<0.05); ** = Highly significant (P<0.01)**

**Table 9.** Association of behavioral habits with different health status groups among respondents

| **Behavioral Habits** | **Groups** | | | | | | **Chi-square**  **(P-value)** |
| --- | --- | --- | --- | --- | --- | --- | --- |
|  | **Normal (%) n=100** | **Diabetic (%) n=100** | | **Cancerous (%) n=100** | **Diabetic Cancerous (%) n=100** | **Total (%) n=400** |  |
| **Physical activity** | | | | | | | |
| Sedentary | 60 (60%) | 69 (69%) | | 85 (85%) | 88 (88%) | 302 (75.5%) | 49.47^**^  P**<0.0001** |
| Mild | 28 (28%) | 31 (31%) | | 13 (13%) | 12 (12%) | 84 (21%) |  |
| Moderate | 11 (11%) | 0 (0%) | | 2 (2%) | 0 (0%) | 13 (3.2%) |  |
| Extremely active | 1 (1%) | 0 (0%) | | 0 (0%) | 0 (0%) | 1 (0.2%) |  |
| **Hours of sleep at night** | | | | | | | |
| 3-4 hours | 16 (16%) | 15 (15%) | | 35 (35%) | 25 (25%) | 91 (22.8%) | 23.77^**^  P**<**0.001 |
| 5-6 hours | 26 (26%) | 28 (28%) | | 35 (35%) | 30 (30%) | 119 (29.8%) |  |
| 7-8 hours | 58 (58%) | 57 (57%) | | 30 (30%) | 45 (45%) | 190 (47.5%) |  |
| **Screen time** | | | | | | | |
| ≤ 2 hours | 59 (59%) | 88 (88%) | | 83 (83%) | 79 (79%) | 309 (77.2%) | 34.94^**^  P**<0.0001** |
| 3-5 hours | 23 (23%) | 6 (6%) | | 12 (12%) | 15 (15%) | 56 (14%) |  |
| 6-8 hours | 9 (9%) | 0 (0%) | | 3 (3%) | 2 (2%) | 14 (3.5%) |  |
| ≥ 9 hours | 9 (9%) | 6 (6%) | | 2 (2%) | 4 (4%) | 21 (5.2%) |  |
| **Stress level** | | | | | | | |
| Mild | 68 (68%) | 43 (43%) | | 65 (65%) | 67 (67%) | 243 (60.8%) | 29.47^**^  P**<0.0001**) |
| Moderate | 15 (15%) | 45 (45%) | | 25 (25%) | 19 (19%) | 104 (26%) |  |
| Severe | 17 (17%) | 12 (12%) | | 10 (10%) | 14 (14%) | 53 (13.2%) |  |
| **Hair dye usage** | | | | | | | |
| Never | 50 (50%) | 23 (23%) | | 28 (28%) | 30 (30%) | 131 (32.8%) | 105.04^**^  P**<0.0001** |
| Used to dye | 0 (0%) | 5 (5%) | | 26 (26%) | 42 (42%) | 73 (18.2%) |  |
| Occasionally | 24 (24%) | 49 (49%) | | 35 (35%) | 21 (21%) | 129 (32.2%) |  |
| Monthly | 26 (26%) | 23 (23%) | | 11 (11%) | 7 (7%) | 67 (16.8%) |  |
| **Facewash usage** | | | | | | | |
| Never | 82 (82%) | 82 (82%) | | 78 (78%) | 87 (87%) | 329 (82.2%) | 10.85^NS^  P**>**0.286 |
| Used to wash | 0 (0%) | 0 (0%) | | 3 (3%) | 2 (2%) | 5 (1.2%) |  |
| Occasionally | 9 (9%) | 13 (13%) | | 12 (12%) | 8 (8%) | 42 (10.5%) |  |
| Regularly | 9 (9%) | 5 (5%) | | 7 (7%) | 3 (3%) | 24 (6%) |  |
| **Deodorant usage** | | | | | | | |
| Never | 92 (92%) | 97 (97%) | | 92 (92%) | 97 (97%) | 378 (94.5%) | 25.24^**^  P**<**0.003 |
| Used to | 0 (0%) | 0 (0%) | | 2 (2%) | 1 (1%) | 3 (0.8%) |  |
| Occasionally | 2 (2%) | 3 (3%) | | 6 (6%) | 2 (2%) | 13 (3.2%) |  |
| Regularly | 6 (6%) | 0 (0%) | | 0 (0%) | 0 (0%) | 6 (1.5%) |  |
| **Supplements usage** | | | | | | | |
| Never | 49 (49%) | | 44 (44%) | 40 (40%) | 30 (30%) | 163 (40.8%) | 10.801^NS^  P>0.290 |
| Former | 10 (10%) | | 10 (10%) | 15 (15%) | 12 (12%) | 47 (11.8%) |  |
| Occasionally | 21 (21%) | | 18 (18%) | 20 (20%) | 25 (25%) | 83 (21.0%) |  |
| Regularly | 20 (20%) | | 28 (28%) | 25 (25%) | 33 (33%) | 106 (26.5%) |  |
| **Smoking** | | | | | | | |
| Never | 79 (79%) | | 96 (96%) | 97 (97%) | 96 (96%) | 368 (92%) | 40.11^**^  P**<0.0001** |
| Former | 5 (5%) | | 0 (0%) | 0 (0%) | 0 (0%) | 5 (1.2%) |  |
| Occasionally | 3 (3%) | | 0 (0%) | 3 (3%) | 1 (1%) | 7 (1.8%) |  |
| Regularly | 13 (13%) | | 4 (4%) | 0 (0%) | 3 (3%) | 20 (5%) |  |
| **Drugs use** | | | | | | | |
| Never | 98 (98%) | 100 (100%) | | 100 (100%) | 100 (100%) | 398 (99.5%) | 6.03^NS^  P**>**0.420 |
| Former | 1 (1%) | 0 (0%) | | 0 (0%) | 0 (0%) | 1 (0.2%) |  |
| Occasionally | 1 (1%) | 0 (0%) | | 0 (0%) | 0 (0%) | 1 (0.2%) |  |
| Regularly | 0 (0%) | 0 (0%) | | 0 (0%) | 0 (0%) | 0 (0%) |  |
| **Vape** | | | | | | | |
| Never | 98 (98%) | | 100 (100%) | 100 (100%) | 100 (100%) | 398 (99.5%) | 6.03^NS^  P**>**0.110 |
| Former | 0 (0%) | | 0 (0%) | 0 (0%) | 0 (0%) | 0 (0%) |  |
| Occasionally | 0 (0%) | | 0 (0%) | 0 (0%) | 0 (0%) | 0 (0%) |  |
| Regularly | 2 (2%) | | 0 (0%) | 0 (0%) | 0 (0%) | 2 (0.5%) |  |
| **Chewing tobacco/Snuff** | | | | | | | |
| Never | 96 (96%) | | 100 (100%) | 100 (100%) | 98 (98%) | 394 (98.5%) | 7.45^NS^  P**>**0.282 |
| Former | 2 (2%) | | 0 (0%) | 0 (0%) | 1 (1%) | 3 (0.8%) |  |
| Occasionally | 0 (0%) | | 0 (0%) | 0 (0%) | 0 (0%) | 0 (0%) |  |
| Regularly | 2 (2%) | | 0 (0%) | 0 (0%) | 1 (1%) | 3 (0.8%) |  |
| **Alcohol consumption** | | | | | | | |
| Never | 98 (98%) | | 100 (100%) | 100 (100%) | 100 (100%) | 398 (99.5%) | 6.03^NS^  P**>**0.110 |
| Former | 2 (2%) | | 0 (0%) | 0 (0%) | 0 (0%) | 2 (0.5%) |  |
| Occasionally | 0 (0%) | | 0 (0%) | 0 (0%) | 0 (0%) | 0 (0%) |  |
| Regularly | 0 (0%) | | 0 (0%) | 0 (0%) | 0 (0%) | 0 (0%) |  |

**NS = Non-significant (P>0.05); * = Significant (P<0.05); ** = Highly significant (P<0.01)**

**Table 10.** Association of environmental factors with different health status groups among respondents

| **Environmental Factors** | **Groups** | | | | | **Chi-square**  **(P-value)** |
| --- | --- | --- | --- | --- | --- | --- |
|  | **Normal (%) n=100** | **Diabetic (%) n=100** | **Cancerous (%) n=100** | **Diabetic Cancerous (%) n=100** | **Total (%) n=400** |  |
| **Exposure to gases, stone dust, coal dust, wood dust, textile dust or fumes of any sort at work** | | | | | | |
| Never | 95 (95%) | 98 (98%) | 98 (98%) | 98 (98%) | 389 (97.2%) | 14.94^NS^  P**>**0.245 |
| ≤ 2 years | 2 (2%) | 0 (0%) | 0 (0%) | 0 (0%) | 2 (0.5%) |  |
| 3-5 years | 0 (0%) | 0 (0%) | 0 (0%) | 1 (1%) | 1 (0.2%) |  |
| ≥ 5 years | 1 (1%) | 0 (0%) | 2 (2%) | 0 (0%) | 3 (0.8%) |  |
| Currently | 2 (2%) | 2 (2%) | 0 (0%) | 1 (1%) | 5 (1.2%) |  |
| **Exposure to radiation, x-ray chemicals, solvents or oil products at work** | | | | | | |
| Never | 95 (95%) | 100 (100%) | 96 (96%) | 97 (97%) | 388 (97%) | 25.48^*^  P**<**0.013 |
| ≤ 2 years | 0 (0%) | 0 (0%) | 0 (0%) | 3 (3%) | 3 (0.8%) |  |
| 3-5 years | 1 (1%) | 0 (0%) | 0 (0%) | 0 (0%) | 1 (0.2%) |  |
| ≥ 5 years | 2 (2%) | 0 (0%) | 0 (0%) | 0 (0%) | 2 (0.5%) |  |
| Currently | 2 (2%) | 0 (0%) | 4 (4%) | 0 (0%) | 6 (1.5%) |  |
| **Exposure to secondhand smoke** | | | | | | |
| Never | 49 (49%) | 59 (59%) | 67 (67%) | 64 (64%) | 239 (59.8%) | 14.91^NS^  P**>**0.094 |
| Used to exposed | 6 (6%) | 0 (0%) | 5 (5%) | 6 (6%) | 17 (4.2%) |  |
| Occasionally | 24 (24%) | 23 (23%) | 14 (14%) | 16 (16%) | 77 (19.2%) |  |
| Regularly | 21 (21%) | 18 (18%) | 14 (14%) | 14 (14%) | 67 (16.8%) |  |

**NS = Non-significant (P>0.05); * = Significant (P<0.05); ** = Highly significant (P<0.01)**

**Table 11.** Association of female-specific characteristics with different health status groups among respondents

| **Parameters** | **Groups** | | | | | **Chi-square**  **(P-value)** |
| --- | --- | --- | --- | --- | --- | --- |
|  | **Normal (%) n=87** | **Diabetic (%) n=100** | **Cancerous (%) n=96** | **Diabetic Cancerous (%) n=51** | **Total (%) n=334** |  |
| **Current menstruation status** | | | | | | |
| Still menstruating | 31 (60.8%) | 44 (50.6%) | 39 (39%) | 8 (8.3%) | 122 (36.5%) | 63.64^**^  P**<0.0001** |
| In menopause | 0 (0%) | 5 (5.7%) | 0 (0%) | 2 (2.1%) | 7 (2.1%) |  |
| Post-menopause | 20 (39.2%) | 38 (43.7%) | 61 (61%) | 86 (89.6%) | 205 (61.4%) |  |
| **PCOS** | | | | | | |
| Yes | 7 (13.7%) | 16 (18.4%) | 11 (11%) | 12 (12.5%) | 46 (13.8%) | 2.34^NS^  P**>**0.505 |
| No | 44 (86.3%) | 71 (81.6%) | 89 (89%) | 84 (87.5%) | 288 (86.2%) |  |
| **Ever taken oral contraceptives** | | | | | | |
| Yes | 6 (11.8%) | 2 (2.3%) | 15 (15%) | 10 (10.4%) | 33 (9.9%) | 8.79^*^  P**<**0.032 |
| No | 45 (88.2%) | 85 (97.7%) | 85 (85%) | 86 (89.6%) | 301 (90.1%) |  |
| **Gestational diabetes** | | | | | | |
| Yes | 0 (0%) | 12 (13.8%) | 2 (2%) | 2 (2.1%) | 16 (4.8%) | 21.28^**^  P**<0.0001** |
| No | 51 (100%) | 75 (86.2%) | 98 (98%) | 94 (97.9%) | 318 (95.2%) |  |
| **Makeup use** | | | | | | |
| Never | 18 (35.3%) | 33 (37.9%) | 29 (29%) | 29 (30.2%) | 109 (32.6%) | 54.49^**^  P**<0.0001** |
| Used to | 3 (5.9%) | 12 (13.8%) | 19 (19%) | 36 (37.5%) | 70 (21%) |  |
| Occasionally | 25 (49%) | 42 (48.3%) | 52 (52%) | 31 (32.3%) | 150 (44.9%) |  |
| Regularly | 5 (9.8%) | 0 (0%) | 0 (0%) | 0 (0%) | 5(1.5%) |  |

**NS = Non-significant (P>0.05); * = Significant (P<0.05); ** = Highly significant (P<0.01**
