# Supplementary material for: Association of diabetes mellitus and breast cancer in adult men and women: a cross-sectional survey
Source: BMC Cancer. 2025 Aug 7;25:1276. doi: 10.1186/s12885-025-14689-6 (PMC12329997; doi:10.1186/s12885-025-14689-6)
Supplement: Supplementary file 3 — Supplementary Material 3 [file 12885_2025_14689_MOESM3_ESM.pdf]

SURVEY QUESTIONNAIRE

Name		Age	☐ 30-40 ☐ 41-50 ☐ 51-60		
Gender	☐ M ☐ F	Height		Weight	
BMI	☐ Underweight ☐ Normal weight ☐ Overweight ☐ Obese				
Fat accumulation area	☐ Abdomen ☐ Breast ☐ Buttocks ☐ Thighs ☐ Shoulders ☐ No				

FAMILY MEDICAL HISTORY

Family history of diabetes	☐ Yes ☐ No<br>If yes, then mention the relation with the member ________
Family history of cancer	☐ Yes ☐ No<br>If yes, then mention the relation with the member ________

PERSONAL MEDICAL HISTORY

Diagnosed with Diabetes	☐ Yes ☐ No
Age when diabetes diagnosed	☐ 18-28 y ☐ 29-38 y ☐ 39-48 y ☐ 49-60 y
Diagnosed with cancer	☐ Yes ☐ No<br>-If yes, specify the type of cancer ________
Age when cancer diagnosed	☐ 18-28 y ☐ 29-38 y ☐ 39-48 y ☐ 49-60 y
Stage of cancer when diagnosed	☐ Stage 0 ☐ Stage I ☐ Stage II ☐ Stage III ☐ Stage IV
Now, stage of cancer	☐ Stage 0 ☐ Stage I ☐ Stage II ☐ Stage III ☐ Stage IV
Metastasis	☐ Yes ☐ No
Any other disease	☐ Yes ☐ No<br>-If yes, then mention ________

DIETARY HISTORY

Food Items	Yes	Never	Occasionally	Used to eat/cook
Fish	_____ times in a week			
Red meat	_____ times in a week			
Processed meat	_____ times in a week			
Meat or poultry	_____ times in a week			
Egg	_____ times in a week			
Fast food (pizza, burger, fries etc.)	_____ times in a week			
Noodle	_____ times in a week			
Pickle	_____ times in a week			
Bakery products	_____ times in a week			
White bread	_____ times in a week			
Homemade butter	_____ times in a week			
Blue band butter	_____ times in a week			
Whole milk	_____ times in a week			
Coffee	_____ times in a week			
Tea	_____ times in a week			
Tea whitener	_____ times in a week			
Soft drinks	_____ times in a week			
Processed fruit-juice	_____ times in a week			
Citrus fruit juices	_____ times in a week			
Fruits	_____ times in a week			
Root vegetables (shljm, chukndr, adrk, alo)	_____ times in a week			
Leafy vegetables (gobi, plk, salad ky pty)	_____ times in a week			
Lentils	_____ times in a week			
White rice	_____ times in a week			
White flour (maida)	_____ times in a week			
Nuts	_____ times in a week			

White sugar	______ times in a week			
Ice cream	______ times in a week			
Chocolate	______ times in a week			
Deep-fried food items	______ times in a week			
Artificial food color	______ times in a week			
Aluminum foils are used for cooking	______ times in a week			
Aluminum utensils are used for cooking	☐ Yes ☐ No			
Microwave usage	☐ Never ☐ Used to ☐ Occasionally ☐ Regularly			
Overcooked food	☐ Yes ☐ No			
Loss of appetite	☐ Yes ☐ No			
Increase in appetite	☐ Yes ☐ No			
Salt intake	☐ 1-2 spoons ☐ 2-4 spoons ☐ 5-6 spoons			
Use for cooking	☐ Oil ☐ Ghee			
Repeatedly use the same oil for frying	☐ Yes ☐ No			
Avoid fat while eating meat	☐ Yes ☐ No			
Follow any specific diet	☐ No ☐ Mediterranean diet ☐ Vegetarian diet ☐ Vegan diet ☐ Ketogenic diet ☐ Non-vegetarian ☐ Other			
**HABITS**				
Physical activity	☐ Sedentary ☐ Mild ☐ Moderate ☐ Extremely active			
Hours of sleep at night	☐ 3-4 hours ☐ 5-6 hours ☐ 7-8 hours			
Stress	☐ Mild ☐ Moderate ☐ Severe			
Smoking	☐ Never ☐ Former ☐ Occasionally ☐ Current smoker			
Drugs use	☐ Never ☐ Former ☐ Occasionally ☐ Current user			
Vape	☐ Never ☐ Former ☐ Occasionally ☐ Current user			
Chew tobacco/snuff	☐ Never ☐ Former ☐ Occasionally ☐ Current user			
Alcohol consumption	☐ Never ☐ Former ☐ Occasionally ☐ Current drinker			
**MISCELLANEOUS INFORMATION**				
Exposure to gases, stone dust, coal dust, wood dust, textile dust or fumes of any sort in your work	☐ Never ☐ ≤ 2 years ☐ 3-5 years ☐ ≥ 5 years ☐ Currently			
Exposure to radiation, X-ray chemicals, solvents or oil products in your work	☐ Never ☐ ≤ 2 years ☐ 3-5 years ☐ ≥ 5 years ☐ Currently			
Screen time	☐ ≤ 2 hours ☐ 3-5 hours ☐ 6-8 hours ☐ ≥ 9 hours			
Exposure to secondhand smoke	☐ Never ☐ Used to exposed ☐ Occasionally ☐ Currently			
Ever used a hair dye	☐ Never ☐ Used to dye ☐ Occasionally ☐ Monthly			
Ever used mouthwash	☐ Never ☐ Used to wash ☐ Occasionally ☐ Regularly			
Ever used deodorant	☐ Never ☐ Used to ☐ Occasionally ☐ Regularly			
Taking supplements	☐ Never ☐ Former ☐ Occasionally ☐ Regularly			
**FOR FEMALES**				
Unusual bleeding from the vagina	☐ Yes ☐ No			
Unusual discharge from the vagina	☐ Yes ☐ No			
Current menstruation status	☐ Still menstruating ☐ In menopause ☐ Postmenopause			
PCOS	☐ Yes ☐ No			
Ever taken oral contraceptives	☐ Yes ☐ No			
Gestational diabetes	☐ Yes ☐ No			
Makeup use	☐ Never ☐ Used to ☐ Occasionally ☐ Regularly			
